# Phytochemicals Target Multiple Metabolic Pathways in Cancer

**DOI:** 10.3390/antiox12112012

**Published:** 2023-11-17

**Authors:** Oleg Shuvalov, Yulia Kirdeeva, Alexandra Daks, Olga Fedorova, Sergey Parfenyev, Hans-Uwe Simon, Nickolai A. Barlev

**Affiliations:** 1 Institute of Cytology of the Russian Academy of Sciences, St. Petersburg 194064, Russia; yulia.kirdeeva@yandex.ru (Y.K.); alexandra.daks@gmail.com (A.D.); fedorovaolga0402@gmail.com (O.F.);; 2 Institute of Pharmacology, University of Bern, 3010 Bern, Switzerland; hans-uwe.simon@unibe.ch; 3 Institute of Fundamental Medicine and Biology, Kazan Federal University, Kazan 420008, Russia; 4 Department of Biomedical Sciences, School of Medicine, Nazarbayev University, Astana 20000, Kazakhstan

**Keywords:** cancer metabolism, metabolic reprogramming and plasticity, natural compounds, multitarget agents, glycolysis, glutaminolysis, one-carbon metabolism, lipid metabolism, β-oxidation of fatty acids

## Abstract

Cancer metabolic reprogramming is a complex process that provides malignant cells with selective advantages to grow and propagate in the hostile environment created by the immune surveillance of the human organism. This process underpins cancer proliferation, invasion, antioxidant defense, and resistance to anticancer immunity and therapeutics. Perhaps not surprisingly, metabolic rewiring is considered to be one of the “Hallmarks of cancer”. Notably, this process often comprises various complementary and overlapping pathways. Today, it is well known that highly selective inhibition of only one of the pathways in a tumor cell often leads to a limited response and, subsequently, to the emergence of resistance. Therefore, to increase the overall effectiveness of antitumor drugs, it is advisable to use multitarget agents that can simultaneously suppress several key processes in the tumor cell. This review is focused on a group of plant-derived natural compounds that simultaneously target different pathways of cancer-associated metabolism, including aerobic glycolysis, respiration, glutaminolysis, one-carbon metabolism, de novo lipogenesis, and β-oxidation of fatty acids. We discuss only those compounds that display inhibitory activity against several metabolic pathways as well as a number of important signaling pathways in cancer. Information about their pharmacokinetics in animals and humans is also presented. Taken together, a number of known plant-derived compounds may target multiple metabolic and signaling pathways in various malignancies, something that bears great potential for the further improvement of antineoplastic therapy.

## 1. Introduction

According to World Health Organization statistics, cancer is the second leading cause of death worldwide (https://www.who.int/health-topics/cancer, accessed on 24 October 2023). According to the Global Cancer Statistics (GLOBOCAN) [1], 19.3 million new cancer cases and almost 10 million cancer-related deaths occurred in 2020 worldwide. Thus, in addition to already established traditional approaches to the treatment of malignancies, there is a constant need to search for and develop new anticancer therapeutics to address this challenge.

To treat malignancies, we need to target those particular characteristics that distinguish neoplastic cells from their normal counterparts. This is required to minimize off-target effects and to protect healthy tissues and organs from the impact of harmful chemotherapeutics.

One of the “Hallmarks of cancer”, which is extensively recognized today in the context of therapy, is metabolic reprogramming and plasticity [2]. Neoplastic cells of different origins are characterized by a set of metabolic alterations and plasticity, which provide malignant cells with energy and adaptational plasticity. These specific metabolic features are suitable targets for therapeutic intervention [3,4].

It is widely accepted today that the inhibition of only one of the processes in a tumor cell, even by a highly specific drug, often leads to a limited response and subsequently to the emergence of resistance [5]. To increase the overall effectiveness of antineoplastic therapy, it is advisable to use several, or multitarget, drugs that can simultaneously suppress several key processes in tumor cells. However, the majority of modern antineoplastic synthetic drug targets are associated with adverse reactions and multidrug tolerance/resistance.

**Figure 1:**
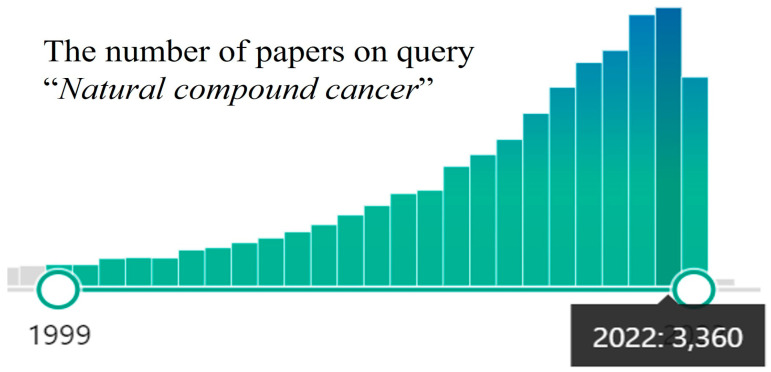
PubMed papers statistics on query “Natural compound cancer” (the query on 19 October 2023).

In the last decade, a surge of interest in using the medicinal potential of natural compounds against cancer has been detected [6,7,8], and the number of publications is constantly growing (Figure 1).

Indeed, natural compounds, along with synthetic chemicals, may be useful to treat malignancies. This approach is well justified because the most frequently used chemotherapeutics are derived from plants and actinomycetes: paclitaxel, vincristine, vinblastine, doxorubicin, camptothecin, etoposide, topo- and irinotecan, etc. For example, our planet harbors about 391,000 plant species, which produce tens of thousands of chemical compounds with a wide range of biological activities, including antineoplastic ones. Moreover, it provides a plethora of candidate compounds with a wide range of sources and novel structures [7].

Secondly, a number of natural compounds from the class of nutraceuticals can kill cancer cells and help anticancer treatment as part of modern chemotherapeutic regimes [9,10,11].

Thirdly, several nutraceuticals like curcumin [12], resveratrol [13], quercetin [14], ginsenosides [15], 20-hydroxyecdysone [16,17], and others not only possess multiple antineoplastic activities but also exhibit pharmacological features (antioxidant, antidiabetic, anti-inflammatory, hepato- and neuroprotective, etc.), which are highly beneficial to cancer patients undergoing chemotherapy:

Traditional Chinese medicine, Ayurveda, Kampo, and other traditional medical systems use herbs and formulations empirically defined over the centuries, which have proven to be effective in preclinical and clinical investigations. In-depth studies of substances widely used in ethnomedicine led to the isolation of a number of compounds with useful biological properties, including antitumor ones [7]. Some of them we frequently consume in the form of food, beverages, spices, or dietary supplements.

Several dozens of natural compounds that target metabolic reprogramming are known and are summarized in a number of excellent reviews [18,19,20].

However, here we review a set of natural compounds that simultaneously meet three criteria: (1) suppress several cancer-associated metabolic pathways; (2) there is information about their pharmacokinetics and bioavailability; (3) the compound generally displays a safety profile or/and has been consumed by people or used in multiple clinical trials.

Malignant cells possess great adaptational plasticity and may adapt to the inhibition of certain biochemical or signaling pathways via the fine-tuning bypass and anaplerotic pathways. Thus, we have focused only on a group of plant-derived natural compounds that simultaneously target different aspects of metabolic reprogramming, including aerobic glycolysis, respiration, glutaminolysis, one-carbon metabolism, de novo lipogenesis, and beta-oxidation of fatty acids.

To make a survey, we collected information from the commonly available databases (MEDLINE/PubMed, Google Scholar, Web of Science, Scopus, Elsevier, SpringerLink, and Wiley Online Library).

Beyond their negative impact on several biochemical pathways, all of the compounds reviewed also target different signaling pathways, including PI3K/AKT/mTOR, ERK/MAPK, Jac/STAT, etc. These properties enhance their multitargeting capacity, which may increase the efficiency of antineoplastic therapy. Moreover, many of them also possess other beneficial pharmacological properties, including antioxidant, hypoglycemic, and hepato- and neuroprotective, which can be extremely useful upon chemotherapeutic intervention to decrease its harmful consequences on non-cancer tissues. As is very important for translational medicine, the safety and bioavailability of reviewed compounds are also discussed.

## 2. Metabolic Reprogramming in Cancer

As mentioned above, metabolic reprogramming is considered to be one of the “Hallmarks of cancer” [2]. To address it as a multitarget for natural compounds, below, we briefly discuss the main attributes of metabolic rewiring and the role of oncogenes and signaling pathways in this complex phenomenon.

### 2.1. Increased Glycolysis (“Warburg Effect”)

Deregulated glycolysis is an important “Hallmark of cancer” [21] and is also known as the “Warburg” effect. The latter implies that cancer cells maintain high levels of glycolysis even under normoxic conditions [22]. This means that various neoplasia utilize glucose more than normal cells due to increased expression of glucose transporters (e.g., GLUT1) and a number of glycolytic enzymes [23].

Enhanced glycolysis is so common in neoplasia that it formed a basis for the approach to detect both primary and secondary tumors in the body by PET/CT. 18F-Fluorodeoxyglucose (FDG) is a glucose analog that is transported via glucose transporters into the cancer cells followed by hexokinase 2 (HK2)-mediated phosphorylation [24,25,26]. Thus, the areas of malignant growth are detected based on the increase in glucose uptake and utilization.

As mentioned above, glucose is transported into malignant cells through a number of glucose transporters (GLUTs); GLUT1 and GLUT3 are considered major ones. These transporters, especially GLUT1, are often up-regulated in different neoplasia and promote their aggressiveness and resistance to therapy [27,28]. The inhibition of these transporters, which may dampen uncontrolled glycolysis, is a developing antineoplastic approach with a number of compounds that are under investigation in preclinical and clinical models [29].

**Figure 2:**
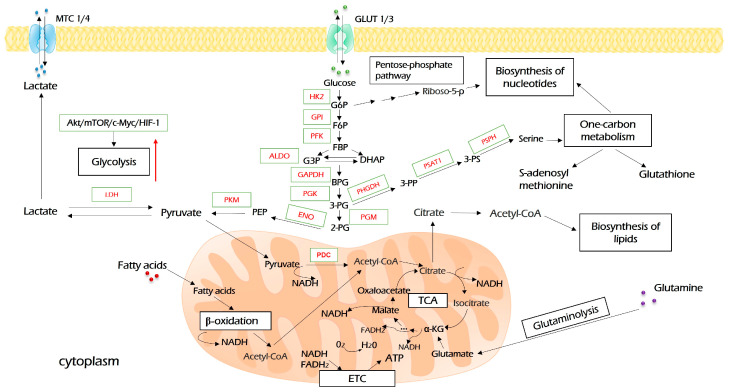
Glycolysis and its interconnection with other metabolic processes. Glucose enters cancer cells through glucose transporters (GLUT1/3) and is oxidized into pyruvate during glycolysis. Pyruvate is further processed in the Krebs cycle (TCA) to produce reducing equivalents (NADH and FADH_2_), which will use the electron transport chain (ETC) to produce ATP upon oxidative phosphorylation. The excessive amount of pyruvate is converted to lactate and exported by monocarboxylate carriers (MCT1/4). Intermediates of glycolysis, G6P, and 3-PG open pentose phosphate pathways and aid in the biosynthesis of serine, which both feed one-carbon metabolism, including the biosynthesis of nucleotides. TCA-derived citrate is re-converted to acetyl-coenzyme A (acetyl-CoA), which is a source of biosynthesis for fatty acids. Further explanations are provided in the text. HK2—hexokinase 2; GPI—glucose-6-phosphate isomerase; PFK—phosphofructokinase; ALDO—aldolase; GAPDH—glyceraldehyde 3-phosphate dehydrogenase; PGK—phosphoglycerate kinase; PGM—phosphoglycerate mutase; ENO—enolase; PKM—pyruvate kinase M; LDH—lactate dehydrogenase; PHGDH—phosphoglycerate dehydrogenase; PSAT1—phosphoserine aminotransferase 1; PSPH—phosphoserine phosphatase.

Upon entering the cell, glucose undergoes a set of enzymatic reactions to form two molecules of pyruvate as the end product (Figure 2). In the first reaction, glucose is activated by hexokinase (HK)-derived phosphorylation. This is the first rate-limiting step of glycolysis. Furthermore, besides glycolysis, the product of this reaction, glucose-6-phosphate, is metabolized in glycogenic, pentose phosphate, and hexosamine biosynthesis pathways, which means that it plays key roles in ATP synthesis, glucose storage, NADH pool enrichment, and protein glycosylation, respectively [30]. Among four HK isoforms identified to date, the oncogenic role of HK2 is widely recognized. Besides its key role in glycolysis, HK2 may be associated with a voltage-dependent anion channel (VDAC) on the outer mitochondria membrane, where it inhibits the activity of pro-apoptotic proteins of the Bcl-2 family and protects tumor cells from death stimuli [31]. All in all, HK2 plays a critical role in oncogenesis in several ways and is a desirable drug target for cancer therapy [30,32].

The second rate-limiting step of glycolysis is mediated by phosphofructokinase (PFK), which catalyzes the conversion of fructose 6-phosphate to fructose 1,6-bisphosphate. PFK1 has three tissue-specific isoforms: platelet (PFKP), muscle (PFKM), and liver (PFKL), all of which may be overexpressed in various malignancies [33,34,35]. In addition to very important metabolic functions in tumor cells, PFK is involved in several signaling pathways, e.g., supporting PI3K, YAP/TAZ, and β-catenin signaling [35,36].

The third rate-limiting glycolytic step is catalyzed by pyruvate kinase (PK). The most prominent isoform with respect to cancer is PKM2. This enzyme catalyzes the last glycolytic step and undergoes a complex allosteric regulation. PKM2 coordinates carbon flux between glycolysis, oxidative phosphorylation, one-carbon metabolism, and glutaminolysis [37,38,39]. Beyond metabolism, PKM2 drives tumorigenesis and chemoresistance by multiple mechanisms, including the activation of HIF1α, c-Myc, STAT3, and Oct-4 [40,41,42,43]. Perhaps not surprisingly, the pharmacologic inhibition of PKM2 is highly desirable and is a subject of many clinical trials.

Further, in normal cells, pyruvate is imported into mitochondria and enters the TCA cycle to support oxidative phosphorylation (OXPHOS). In contrast, in cancer cells, an excessive amount of pyruvate is synthesized due to the significant up-regulation of glycolysis, which may slow down this process. In this case, the excessive pyruvate is converted to lactate by lactate dehydrogenases (LDH) [44]. High glucose uptake and lactate production are two well-known hallmarks of cancer metabolism.

All LDH isoforms, but especially LDHA, promote diverse malignant properties and drive key oncogenic processes [45]. They increase cancer-associated metabolic changes, enhance growth, metastatic potential, and resistance to therapy, diminish antitumor immunity, etc. The high serum level of LDH activity is well known among oncologists as a robust marker of poor prognosis and response to therapy [46].

Itself, lactate is a toxic compound and leads to the acidification of cytosol. Thus, to overcome its toxicity, lactate should be exported outside the cell. This process is mediated by MCT and results in the acidification of intracellular space around tumor cells. Among the four MCT isoforms, MCT1 and MCT4 are the ones predominantly expressed in cancer and have been identified as potential therapeutic targets [47]. Interestingly, MCTs are not only involved in lactate excretion but may also import lactate in OXPHOS-dependent malignant cells, cancer-associated fibroblasts (CAFs), or other cells of tumor microenvironment for its subsequent oxidation, which drives a metabolic symbiosis inside the tumor [48,49]. MCTs are often overexpressed in malignancies; they favor the formation of metastasis and angiogenesis [50,51,52]. The MCTs targeted therapy undergoes preclinical and clinical trials [53].

At first glance, the increased glycolysis in cancer cells seems to be a paradox because glycolysis is not an efficient process for ATP production. In theory, only two ATP molecules are produced per one glucose molecule upon glycolysis, instead of 38 ATP molecules produced by OXPHOS as a continuation of glycolysis. However, there are several reasons for choosing this pathway over OXPHOS, some of which are listed below [54].

Glycolysis allows cancer cells a rapid ATP synthesis. Moreover, it promotes flux into biosynthetic pathways. The intermediate product of glycolysis, 3-phosphoglycerate, can be converted in three steps by PHGDH, PSPH, and PSAT1 to serine, which opens a gate to one-carbon metabolism and biosynthesis of nucleotides. This can be viewed as an anabolic bridge linking glucose assimilation with one-carbon metabolism. Citrate, which is derived from pyruvate in the TCA cycle, is the source for lipogenesis and biosynthesis of several amino acids. Moreover, glycolysis-mediated acidification inactivates the anticancer immune response and fine-tunes the tumor microenvironment. Finally, glycolysis may impact signal transduction cues through its intermediates that possess properties of signaling molecules (for instance, fructose 1,6-bisphosphate [55] and lactate [56]).

Consequently, glycolysis is modulated by a number of oncogenes. Among others, its major regulators are c-Myc, HIF1α, AKT, and mTOR, which may transactivate glycolytic genes (c-Myc, HIF1α) or can directly and indirectly modulate the activity of enzymes through post-translational modifications and protein–protein interactions [57].

Beyond the increased levels of proliferation and metastasis [58,59], high glucose initiates genome instability and de novo mutations, including KRASG12D, in nontumorigenic pancreatic cells [60]. In addition, high glucose may lead to nucleotide imbalance [61] and inhibit nucleotide excision repair (NER) [62].

### 2.2. TCA and OXPHOS

**Figure 3:**
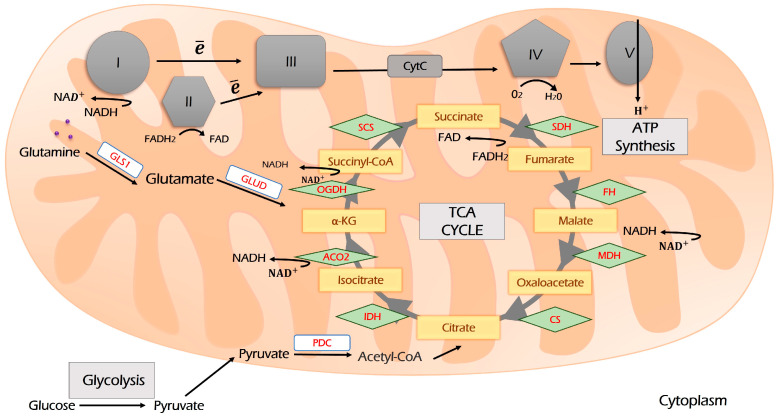
The scheme of the TCA cycle and its coupling with the electron transport chain (ETC). Glycolysis-derived pyruvate enters the TCA cycle through the condensation of acetyl-CoA and oxaloacetate. During the TCA cycle, reducing equivalents NADH and FADH_2_ are produced, which are oxidized by ETC complexes I and II. Glutaminolysis and β-oxidation of fatty acids are two other sources supplying TCA. Further explanations are provided in the text. I, II, III, IV and V—Respiration Complexes I, II, III, IV and V, respectively; TCA—tricarboxylic acid cycle (Krebs cycle); CS—citrate synthase; IDH—isocitrate dehydrogenase; ACO—aconitase; OGDH—oxoglutarate dehydrogenase; SCS—succinyl coenzyme A synthetase; SDH—succinate dehydrogenase; FH—fumarate hydratase; MDH—malate dehydrogenase; PDC—pyruvate dehydrogenase complex; GLS1—glutaminase 1; GLUD—glutamate dehydrogenase.

Pyruvate links glycolysis to respiration. It is imported into mitochondria and oxidized by PDK to acetyl-CoA (Figure 3). Acetyl-CoA is a primary source for both lipogenesis (the process of fat formation) and the Krebs (TCA) cycle to fuel OXPHOS. TCA is a hub that re-distributes carbon sources for generating cellular energy and is a precursor for biosynthetic pathways linking glycolysis, glutaminolysis, biosynthesis, and beta-oxidation of fatty acids, respiration, and amino acids metabolism into the metabolic network (Figure 2). Its intermediates are citrate and α-ketoglutarate (α-KG). α-KG can be derived from glutamine upon glutaminolysis and then may be reductively carboxylated to form citrate, which fuels the TCA cycle and OXPHOS. This process is called anaplerosis [63,64].

Beyond the high catabolic and biosynthetic importance of TCA, several types of malignancies (Acute myeloid leukemia (AML), glioma, paraganglioma, etc.) bear mutations that lead to the dysregulation of one of three TCA enzymes: isocitrate dehydrogenase (IDH), succinate dehydrogenase (SDH), or fumarate hydratase (FH), which, in turn, promotes the synthesis of oncometabolites and favors tumorigenesis in multiple ways [65].

As a result of TCA, NADPH and NADH are produced (Figure 3). They are further oxidized by respiration chain complexes to produce ATP.

In the 1920s, Otto Warburg suggested that the up-regulation of aerobic glycolysis in cancer is the result of mitochondria dysfunction. However, it is widely accepted today that in line with increased glycolysis, mitochondria play a key role in oncogenesis by providing building blocks for tumor anabolism, maintaining redox and calcium homeostasis, and participating in transcriptional and cell death regulation [66]. Cancer cells extensively use TCA and OXPHOS for both biosynthetic and energy production purposes, along with an increased intensity of glycolysis.

Indeed, the contribution of glycolysis to total ATP production in various malignancies ranges from 1 to 64% [67]. Furthermore, the OXPHOS targeting results in growth inhibition, apoptosis, and susceptibility to cytotoxic drugs [67]. Interestingly, the OXPHOS contribution to ATP production in cancer cells can be reduced to approximately 30% under hypoxia.

Furthermore, there is evidence that tumor stem cells derived from brain, pancreatic, lung, and ovary cancer preferentially use OXPHOS to produce ATP [68,69,70]. Moreover, metabolic plasticity, in many ways, depends on the ability to switch between glycolysis and OXPHOS [2,70].

Taken together, this means a complex interaction between glycolysis and OXPHOS in tumorigenesis, which will be discussed later.

### 2.3. Metabolism of Glutamine

Glutamine is the most abundant amino acid in blood and muscles [71]. Moreover, along with glucose, this is the most important source of carbon and nitrogen to neoplastic cells. Malignant cells, alongside rapidly dividing their non-cancer counterparts, display a high dependency on glutamine [71,72].

**Figure 4:**
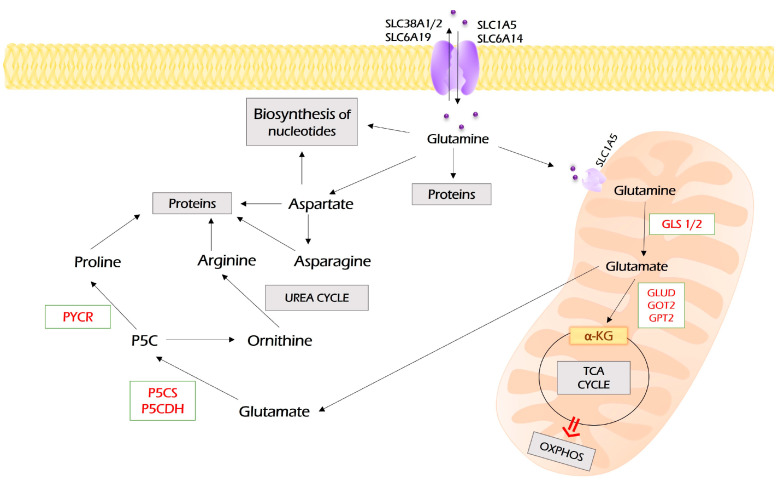
Metabolism of glutamine. Glutamine enters the cell through several membrane transporters depicted. It can be directly used for protein synthesis and as a source for the biosynthesis of nucleotides. In addition, glutamine can be converted into aspartate and further to asparagine. However, a majority of glutamine is imported into mitochondria through SLC1A5, where it undergoes glutaminolysis, the process of its conversion to glutamate by glutaminase (GLS1). Glutamate is a substrate for a set of aminotransferases (glutamate dehydrogenase (GLUD), glutamic oxaloacetic transaminase (GOT2), glutamic pyruvic transaminase 2 (GPT2)), which all may convert it to α-ketoglutarate, the intermediate of the TCA cycle. In addition, glutamate can be exported to the cytoplasm, where it can be used for the synthesis of proline and arginine. PYCR—pyrroline-5-carboxylate reductase; P5CS—pyrroline-5-carboxylate synthase; P5CDH—pyrroline-5-carboxylate dehydrogenase.

Glutamine enters tumor cells by SLC1A5 (ASCT2), SLC38A1, SLC38A2, SLC6A14 (ATB0^+^), and SLC6A19 (B0AT1), which are frequently overexpressed in different malignancies [73] (Figure 4).

The process of glutamine assimilation occurs in mitochondria and is called “glutaminolysis”. Glutamine is transported to mitochondria by the SLC1A5 variant [72]. Glutaminolysis is catalyzed by glutaminase, which is encoded by two isoforms: GLS1 and GLS2. The cancer-associated glutaminolysis is linked to GLS1, which is overexpressed by various neoplasia [71].

Then, glutamate can be converted to α-KG by GLUD (GDH), which, in turn, fuels the TCA cycle and OXPHOS or becomes a substrate for transaminase, including glutamic pyruvic transaminase 2 (GPT2), glutamic oxaloacetic transaminase 2 (GOT2), or PSAT1 to produce non-essential amino acids (alanine, aspartate, and phosphoserine, respectively).

Moreover, glutamate fuels the biosynthesis of glutathione tripeptide, which plays a key role in redox homeostasis and mediates antioxidant defense. In the cytoplasm, glutamine can be converted to aspartate by asparagine synthetase (ASNS) [74]. Finally, both glutamine and glutamine-derived aspartate are carbon and nitrogen donors for the biosynthesis of both pyrimidine and purine nucleotides [71,75].

As with any other metabolic processes altered by cancer, the metabolism of glutamine is controlled by a number of oncogenes and oncosupressors [76]. For example, c-Myc promotes glutamine uptake by transactivating genes coding for glutamine transporters SLC1A5 [77,78]. Furthermore, in enhances the expression of GLS1 via the suppression of its negative regulators: miR-23a, miR-23b [79], and lncRNA GLS-AS [80]. It enhanced the expression of GLUD1, GPT2, GOT1, GOT2, and PSAT1 [76]. mTORC1 up-regulates GLS1 by increasing c-Myc expression [81]. In pancreatic adenocarcinoma, KRAS reprograms glutamine metabolism toward glutamine-derived aspartate synthesis, NADPH production, and balancing cellular redox homeostasis with macromolecular synthesis [82,83].

Thus, glutamine is a prominent carbon and nitrogen donor that supplies both energy production through entering TCA and OXPHOS and fuels biosynthetic processes to produce fatty acids, non-essential amino acids, glutathione, and both pyrimidine and purine nucleotides. The inhibition of GLS1 and GDH and suppression of glutamine metabolism are recognized as an important antineoplastic approach [71,75]. It inhibits cancer growth, metastasis, and mitochondrial respiration and suppresses cancer stem cells [84,85,86].

### 2.4. Lipid Metabolism

In addition to the Warburg phenomenon and increased glutaminolysis, lipid metabolism also undergoes comprehensive metabolic reprogramming in neoplastic cells. Generally, it includes fatty acid (FA) uptake, de novo biosynthesis of lipids (lipogenesis), and fatty acid β-oxidation (FAO) [87]. All of these processes are associated with tumorigenesis and promote proliferation, migration, invasion, and drug resistance of malignant cells and fine-tune their interaction with the microenvironment.

**Figure 5:**
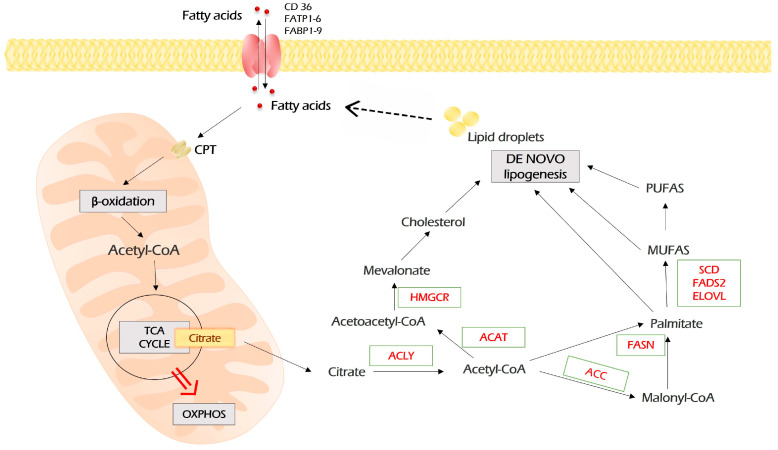
Lipid metabolism in malignant cells. Fatty acids enter malignant cells through a number of importers on the plasma membrane. In addition, in malignant cells, fatty acids can be synthesized de novo through the conversion of citrate, the intermediate of TCA, to acetyl-CoA, which is the precursor of all lipids. In turn, lipids, which are stored as lipid droplets, can be lysed into free fatty acids, which are sources for β-oxidation. In this case, fatty acids are imported into mitochondria by carnitine palmitoyltransferases (CPT 1 and 2), which are localized on the outer and inner mitochondria membrane, respectively. During repeated circles of β-oxidation, fatty acids are broken down to acetyl-CoA monomers, which enter TCA and fuel the energy production by OXPHOS. Further explanations are provided in the text. MUFAs—monounsaturated fatty acids; PUFAs—polyunsaturated fatty acids; FASN—fatty acid synthase; ACC—acetyl-CoA carboxylase; ACLY—ATP citrate lyase; ACAT—acyl-coenzyme A:cholesterol acyltransferases; HMGCR—3-hydroxy-3-methyl-glutaryl-coenzyme A reductase; SCD—stearoyl-CoA desaturase; FADS2—fatty acid desaturase 2; ELOVL—elongation of very long-chain fatty acids protein.

Fatty acids may enter cancer cells by diffusion or by being imported by FA transport proteins (Figure 5). FA transport proteins are represented by FATP1-6 (fatty acid transport protein 1-6), FABP1-9 (fatty acids-binding proteins 1-9), and fatty acids translocase CD36. Inside the cell, fatty acids are reversibly bound to FABPs, which function as intracellular lipid chaperons.

Upon entering the cell, fatty acids undergo an activating conjugation with coenzyme A, which is mediated by acyl-CoA synthetases (ACSS, ACSM, ACSL). Then, for β-oxidation, fatty acyl-CoA needs to get into the mitochondria. This rate-limiting step is catalyzed by carnitine palmitoyltransferases (CPT1 and CPT2) localized on the outer and inner mitochondria membrane, respectively.

In mitochondria, during the β-oxidation process, fatty acyl-CoA is cleaved into acetyl-CoA by a repeated four-step cycle catalyzed by four enzymes. The end product, acetyl-CoA, enters the TCA cycle, which is followed by oxidative phosphorylation to generate ATP [88].

De novo lipogenesis starts with acetyl-CoA, which is a “building block” for all fatty acids. The main source of acetyl-CoA is oxidative decarboxylation of pyruvate, which occurs after glycolysis. In addition, acetyl-CoA can be produced from citrate upon glutaminolysis and β-oxidation of fatty acids. Citrate is converted to acetyl-CoA by ATP citrate lyase (ACLY). Then, acetyl-CoA carboxylase (ACC) catalyzes the transformation of acetyl-CoA and one bicarbonate molecule into malonyl-CoA. Further, fatty acid synthase (FASN) catalyzed the synthesis of palmitate from malonyl-CoA and acetyl-CoA (Figure 5).

The uptake, storage, and use of lipids are an important part of cancer cells’ adaptation for metastasis development [87]. Based on the experimental observations, Lee and colleagues suggested that FAO is the main ATP source in malignant cells of different origins [89]. In addition, it has long been known that in non-glycolytic types of neoplasia like prostate cancer, lymphoma, and pancreatic ductal carcinoma, FAO is the prominent pathway for energy production [90,91,92].

The oncogenic signaling pathway drives rewiring in lipid metabolism [93]. For instance, c-MYC regulates lipogenesis by inducing SREBP1 [94]. Transcriptional coactivator yes-associated protein (YAP) drives metabolic shift toward FAO in lymph node metastasis [95]. Mutant KRAS mediates the reprogramming of lipid metabolism through acyl-coenzyme A (CoA) synthetase long-chain family member 3 (ACSL3) in lung cancer [96]. PI3K/Akt/mTOR axis up-regulates CD36 and SREBP1 and induces lipogenesis [97]. These are only several examples of exploiting lipogenesis by cancer cells. For additional information, please see the excellent reviews on the topic [93,98,99].

Importantly, not only oncogenes affect lipid metabolism. The other way around is also possible, i.e., lipid metabolism can affect oncogenic signaling. The composition of the cell membrane (the profile of FA moieties, content of sphingolipid and cholesterol, etc.) may dramatically affect signaling cascades [87,100]. For instance, the degree of membrane saturation driven by the biosynthesis of several enzymes of fatty acids promotes EGFR clustering and activation of signaling [101,102]. Another example comes from prostate cancer, where polyunsaturated fatty acids modify phospholipid content, which, in turn, alters PIP3/AKT activation [103].

The high lipid diet is closely related to cancer development. In addition, different clinical studies suggest that obesity and the risk of cancer [104,105] are highly associated. Different experiments revealed that a lipid-enriched environment reprograms malignant cells to uptake and metabolize FA to support malignant growth. The increased consumption of lipids drives cancer growth and the development of metastasis in murine breast, colorectal, and gastric cancer models. For instance, the high-fat diet increased CD36 expression and induced metastasis in a gastric cancer mouse model [106]. High fatty acids induced migration and invasion of pancreatic cancer cells and shifted them to oxidative metabolism [107]. In an intriguing study by Lee and colleagues, the tumor growth in the KRAS-mutant mouse tumor model was two times higher in high-fat-consuming mice compared with the control (normally fed) group. However, the tumor growth was three times slower in the low-fat-consuming (but calory balanced) group compared to the control (normal fat diet) [89], suggesting that tumor growth depends on fatty acids as the primary source of energy.

There is much evidence that nearby adipocytes may induce the reprogramming of lipid metabolism in cancer cells [92,100]. The co-cultivation of ovarian cancer cells with adipocytes induced the expression of CD36, an FA receptor, which enhanced the metastatic potential and xenograft growth. CD36 knockdown or use of specific antibodies disrupted this adipocyte-mediated reprogramming [108]. In general, cancer-associated adipocytes imply various mechanisms to promote tumor development. Presumably, this may be an explanation for the fact that many tumors frequently metastasize to adipocyte-rich tissues [92].

Taken together, these observations highlight the notion that the augmentation of lipid uptake may reprogram the metabolism, thereby altering signaling pathways and hence promoting the malignant phenotype and development of metastasis.

## 3. Interplay between Biochemical Pathways Drives Metabolic Plasticity

It is widely accepted now that metabolic rewiring provides selective advantages to cancer cells not by simply deregulating their metabolic pathways but rather by conferring metabolic plasticity, allowing them to switch between different states as part of the adaptation process [109,110]. Unfortunately, the exact molecular mechanisms underpinning metabolic plasticity are far from being completely understood. However, there are numerous reports demonstrating that metabolic plasticity provides cancer cells with energy and “building” blocks required for proliferation, invasion, metastasis, and resistance to the immune system and therapy [2,3].

One of the main features of metabolic rewiring and plasticity is the metabolic heterogeneity of malignant cells, which is a characteristic of many tumors [2]. This feature is well illustrated by the interplay between glycolysis and OXPHOS [67].

An example of such interplay is cancer stem cells (CSCs). CSCs are cells with the ability to self-renew and initiate tumors. They are responsible for cancer recurrence and drug resistance [111,112]. It was previously accepted that CSCs have a more glycolytic phenotype. Indeed, CSCs of different origins, including breast, gastric, and hepatocellular carcinoma, have been reported highly expressing glycolytic genes and have enhanced glycolysis and a low OXPHOS level [70,113,114]. Oppositely, there are a number of reports about OXPHOS-dependent CSCs from glioma, leukemia, ovarian, hepatic, and pancreatic cancer [68,69,115].

Actually, the simultaneous occurrence of both glycolytic and OXPHOS CSCs was reported for pancreatic, breast, and other tumor types [70,116,117].

It has been shown recently that there are two populations of stem cells in isogenic murine glioma, one of which is glycolytic, whereas another one relies on OXPHOS depending on the metabolic characteristics of the tumor cells of origin. The authors report that both phenotypes are independent and stable. However, the OXPHOS population is switched for glycolysis under either hypoxia or metabolic inhibitors [118].

Thus, several authors suggested the existence of a hybrid metabolic state (glycolytic/OXPHOS), which allows malignant cells to switch to the most appropriate metabolic mode under specific conditions in order to facilitate adaptation and survival [119,120,121]. For instance, breast cancer cells are generally characterized by high metabolic heterogeneity [120,122,123]. This allows them to colonize different niches. The lung tissues have high oxygenation, and lung metastasis is derived by OXPHOS cancer cells. In contrast, there is a low oxygen level in hepatic; hepatic metastasis is derived from glycolytic cancer cells [123]. In line with this, it was shown that the loss of GPX2 increased HIF1α expression and glycolytic phenotype while reducing OXPHOS. However, in one specific cell cluster, the loss of GPX2 induced a hybrid phenotype with increased both HIF1α- and AMPK-regulated EMT/stem-like gene signatures [120].

In general, glycolysis-derived pyruvate is the main source of TCA and OXPHOS. However, not only glycolysis fuels respiration. The metabolic plasticity arises from a diversity of mitochondrial metabolites, which may be used as primary energy fuel for OXPHOS under certain conditions (Figure 2). NADH and FADH2 equivalents derived from the metabolic conversion of amino acids and β-oxidation may also be oxidized by mitochondrial respiratory chain complexes [124].

For example, there may be cooperation between glycolytic and OXPHOS cancer cells or between cancer and stromal cells within a tumor [125,126]. In this case, OXPHOS-dependent cells import lactate and convert it to pyruvate, thus fueling TCA and OXPHOS. This phenomenon is called the reversed Warburg effect and has been observed, for instance, in tumor microenvironments, when glycolysis in the cancer-associated stroma metabolically supports adjacent cancer cells [125].

The conversion of serine to glycine in the mitochondrial folate cycle generates a significant amount of NADH2, which may be used by OXPHOS and provides metabolic plasticity for breast cancer cells [124,127].

Proline is oxidized to pyrroline-5-carboxylate by proline carboxylase, which is linked with respiratory chain complexes II and III. This reaction generates FADH2 and supports tumorigenesis and the development of lung metastases in the orthotopic 4T1 and EMT6.5 mouse models [128].

Glutamine derived from glutaminolysis is further converted to aKG, which enters the TCA cycle and supports OXPHOS [129]. In addition, glutamate dehydrogenase (GDH), which is responsible for this last step, is accompanied by the generation of NADH.

Thus, to suppress this complex interplay between glycolysis and OXPHOS, which provides neoplastic cells with adaptational plasticity, survival, and growth advantages, we need to target not only single processes but also the full network and key molecular mediators that govern this metabolic plasticity.

## 4. Oncogenic Signaling Pathways Regulate Metabolic Rewiring and Plasticity

When talking about therapeutic strategies in the context of metabolic rewiring in cancer, a mere inhibition of certain metabolic enzymes is clearly not enough to produce a sustainable therapeutic effect. To develop efficient approaches that target metabolic reprogramming, it is also important to take into consideration the molecular drivers that promote metabolic dysregulation and plasticity.

Different oncogenes and oncosupressors can modulate cancer-related metabolic alterations [2,130,131,132]. The best examples are transcriptional factors c-Myc and HIF1α, two master regulators of glycolysis and other metabolic pathways, which may directly transactivate dozens of metabolic genes [133,134,135].

There is also a complex network of post-translational regulatory circuits of glycolysis mediated by major oncogenes such as AKT, mammalian target of rapamycin (mTOR), epidermal growth factor receptor (EGFR), Kirsten rat sarcoma virus (K-Ras), and others [136,137].

As a master-regulator of anabolic pathways, mTOR drives glycolysis, one-carbon, and lipid metabolism [138]. Its catalytic subunits, mTORC1 and mTORC2, induce the expression of GLUT1 [139] and of the most important mediators of glycolysis—HK2, PFK, and PKM2—through the up-regulation of HIF1α and c-Myc [140,141]; induce the biosynthesis of purine [142] and pyrimidine [143] nucleotides; control biosynthesis and β-oxidation of fatty acids by regulation of SREPB [144,145] and PPARγ [146,147]; and directly phosphorylate and activate ACLY [148]. mTOR-mediated metabolic rewiring confers resistance to chemotherapeutics [149,150].

By multiple mechanisms, including mTOR-dependent or -independent manner, AKT leads to the activation of SREBP, c-Myc, HIF1α, and ATF4 [151]. It directly phosphorylates HK2 [57] and PFKB2 [152] and up-regulates GLUT1 expression [153].

In premalignant pancreatic cells, mutant KRAS drives metabolic reprogramming to induce expression of HK2, LDHA, PDK, glutaminase 1 (GLS1), glutamate dehydrogenase 1 (GLUD1), and transaminases (GOT1, GPT2, and PSAT1), making cells dependent on glucose and glutamine [154]. In addition, KRAS induces GLUT1 and glucose flux to PPP [60].

Both EGFR and Her2 (ErbB family receptors) are drivers of metabolic reprogramming. EGFR and Her2 enhanced glycolysis in triple-negative breast cancer [155,156]. EGFR hyperactivation, either due to its amplification or mutation, elicits metabolic rewiring by activation of the mTORC2/Akt/c-Myc pathway [157]. There is much evidence about the up-regulation of lipogenesis in EGFR-mutated cancer cells resistant to TKI [158]. In EGFR-mutated lung cancer cycling persisted cells, the shift of metabolism toward FAO was observed upon treatment with tyrosine kinase inhibitor [159]. In Her2-overexpressing breast cancer cells, there is usually an enhanced metabolism of glutamine [160,161].

Indeed, the situation is more complex due to a plethora of mi-RNAs, which affect both mRNAs of enzymes and their regulators [162].

## 5. Plant-Derived Compounds Targeting Multiple Biochemical Pathways

### 5.1. Kaempferol

**Figure 6:**
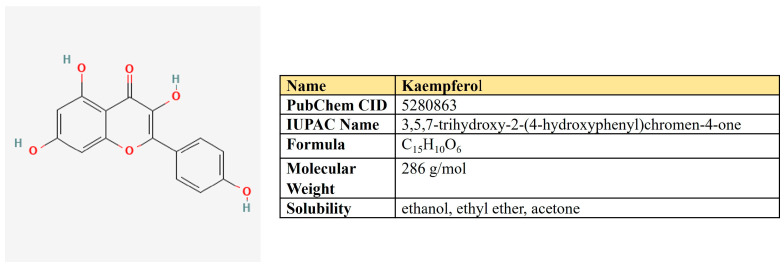
The structure and chemical properties of kaempferol.

Kaempferol is a flavanol first derived from the rhizome of *Kaempferia galanga* (Figure 6). This is a non-toxic, low-price dietary ingredient that is fairly well represented in the daily diet. Kaempferol mitigates inflammatory processes and may reduce osteo- and rheumatoid arthritis, colitis, and gastric ulcer [163].

Dozens of studies have shown its antineoplastic activity (Table 1). Kaempferol inhibits cancer-associated signaling pathways [164], suppresses proliferation, angiogenesis, and migration, and reverses drug resistance [165,166].

One more mechanism of kaempferol antineoplastic activity is linked to its negative impact on metabolic processes. Yao and colleagues have demonstrated kaempferol-mediated EGFR-dependent inhibition of glucose uptake and lactate production in esophagus carcinoma. In this case, kaempferol suppressed EGFR and HK2 both in vitro and in vivo [167].

In addition, kaempferol was shown to inhibit glycolysis in colon cancer through the up-regulation of specific micro-RNAses. Firstly, kaempferol increased the expression of miR-339-5p, which targets hnRNPA1 and PTBP1, which, in turn, produces PKM2 upon splicing [168]. This led to reduced lactic acid and ATP production. Furthermore, kaempferol increased the expression of miR-326 directly targeting PKM2, which was accompanied by the reverse resistance to 5-FU [169].

Another mechanism of kaempferol-mediated inhibition of glycolysis was proposed in melanoma cells. Kaempferol prevented the binding of HK2 and VDAC1 on mitochondria through the AKT/GSK-3β signaling pathway, which suppressed pyruvate and lactate production and metastasis [170].

Besides enzymes of glycolysis, kaempferol is suggested to negatively affect its key transcriptional regulators: HIF1α and c-Myc. Kaempferol glycosides induced ubiquitin-proteasome-dependent degradation of HIF1α, which inhibited hypoxia signaling and expression of GLUT1 in pancreatic cancer cells [171]. In hepatoma cells, kaempferol did not alter the HIF1α protein level but changed its localization by the inactivation of p44/42 MAPK [172]. In addition, two groups of researchers have revealed that kaempferol may bind G-quadruplex in the c-Myc promotor region, thereby suppressing its expression [173,174].

Beyond glycolysis, kaempferol was able to suppress respiration in Hela cells by inhibiting the mitochondrial respiratory chain complex I. This led to a failure of energy and induced autophagy by increased AMPK [175].

In addition, Brusselman and colleagues showed kaempferol-mediated inhibition of FASN and lipogenesis in prostate and breast cancer cells [176].

**Table 1:** Kaempferol-mediated impact on metabolic pathways in cancer models.

Metabolic Pathway Affected	Related Targets	Type of Neoplasia	Description	Reference
Glycolysis	HK2; EGFR	Esophagus carcinoma	Decrease in EGFR, HK2, glucose uptake, and lactate production in vitro and in vivo	[167]
Glycolysis	PKM2	Colon cancer	Increase expression of miR-326, which directly targets PKM2; reverse resistance to 5-FU	[168]
Glycolysis	PKM2	Colon cancer	Increase expression of miR-339-5p, which targets hnRNPA1/PTBP1/PKM2 axis a	[169]
Glycolysis	HK2 and VDAC1	Melanoma	Prevention of HK2 and VDAC1 binding on mitochondria	[170]
Glycolysis	c-Myc	Cervical and colorectal cancer	Binding of kaempferol with G-quadruplex in promotor region; decrease in c-Myc expression	[174]
Glycolysis	HIF1α	Pancreatic cancer	Proteasome-dependent degradation of HIF1α; decrease in GLUT1 expression	[171]
Glycolysis	HIF1α	Hepatic cancer	Inhibition of p44/42 MAPK led to inactivation of HIF1α by its cytoplasmic localization	[172]
OXPHOS	Complex I	Cervical cancer	Inactivation of respiratory chain complex I led to energy failure and AMPK-dependent autophagy	[175]
Fatty acids biosynthesis	FASN	Prostate and breast cancer	Inhibition of FASN and lipogenesis	[176]

**Figure 7:**
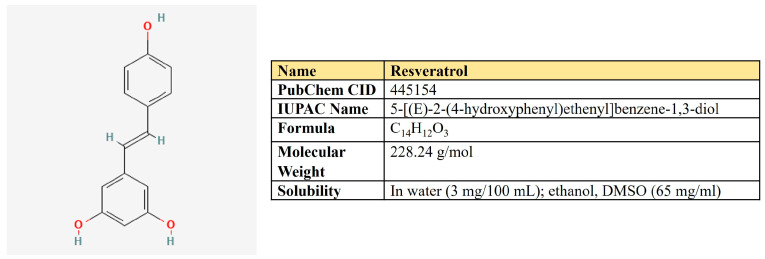
The structure and chemical properties of resveratrol.

***Resveratrol.*** Resveratrol is a 3,5,4′-trihydroxystilbene that consists of two aromatic rings that are connected through a methylene bridge (Figure 7). This is a dietary polyphenol that is present in significant amounts in grapes, wine, peanuts, and berries. It is also consumed as a dietary supplement due to its anticancer, chemopreventive, antiviral, antifungal, anti-aging, and anti-inflammatory activities [177].

The plethora of resveratrol antineoplastic properties is associated with its negative impact on the cell cycle, angiogenesis, and cell signaling pathways, as well as positive modulation of autophagy and apoptosis [178] (Table 2). Different studies also link resveratrol-mediated antineoplastic effects with the activation of p53 oncosupressor (reviewed in [179]).

There are many reports that resveratrol targets glycolysis by various mechanisms in different types of neoplasia. Indeed, resveratrol suppressed EGFR, Akt, and ERK1/2 activation, which led to inhibition of HK2-mediating glycolysis in NSCLC [180]; down-regulated HK2 in hepatocellular carcinoma both in vitro and in vivo [181]. It suppressed glycolysis in pancreatic cancer cells by targeting miR-21 [182].

Regarding the model of angiogenesis, resveratrol down-regulated VEGF-induced glycolysis in human umbilical vein endothelial cells (HUVECs), which was associated with the inhibition of GLUT1, HK2, PFK1, PKM2 expression, and PKM2 mis-localization [183].

Not only glycolytic enzymes but also critical regulators of glycolysis are affected by resveratrol. In ovarian cancer cells, resveratrol suppressed glycolysis, proliferation, and migration. The molecular mechanism behind these effects involves activation of AMPK and, hence, inhibition of mTOR [184]. In colon and breast cancer cells, resveratrol inhibited the expression of c-Myc, VEGF, and hTERT [185]. In Lewis lung carcinoma tumor-bearing mice, resveratrol suppressed the intake of (18)F-FDG, and glycolysis decreased the protein level of HIF1α, Akt, and mTOR [186]. Molecular docking experiments suggested that resveratrol may be a direct inhibitor of HIF1α; it down-regulates its protein level in pancreatic cancer cells [187].

Vanamala and colleagues have applied the proteomic approach to search proteins altered by resveratrol in colon cancer cells. They observed that G6PD and transketolase, two key enzymes of the pentose phosphate pathway (PPP), were down-regulated by this compound, which links resveratrol with the down-regulation of PPP [188].

Another research group has shown that resveratrol suppressed c-Myc, glucose consumption, and glycolytic enzymes PK and LDH. However, it increased citrate synthase, one of the enzymes of the Krebs cycle [189]. In another study on colon cancer cells, the authors have also shown resveratrol-mediated down-regulation of glycolysis while increasing glucose oxidation. These observations were accompanied by down-regulation of PPP and lipogenesis by resveratrol [190]. However, other authors have shown that both glycolysis and respiration have been targeted by resveratrol in Hela cells, including several key OXPHOS proteins [191].

Beyond the metabolism of glucose, resveratrol suppressed the expression of glutamine importer ASCT2 in hepatoma cells, which increased sensitivity to cisplatin [184]. In addition to that, resveratrol may suppress de novo fatty acid biosynthesis. As mentioned above, it activates AMPK and down-regulates mTOR in breast cancer cells, subsequently inhibiting acetyl-CoA carboxylase α (ACACA) and fatty acid synthase (FASN) [192]. In line with this notion, resveratrol was shown to down-regulate FASN in Her2-overexpressing breast cancer [193].

Taken together, it seems that depending on a particular cellular context, resveratrol may target a broad spectrum of metabolic pathways in neoplastic cells, including glycolysis, respiration, pentose phosphate pathway, biosynthesis of fatty acids, and glutamine uptake.

**Table 2:** Resveratrol-mediated impact on metabolic pathways in cancer models.

Metabolic Pathway Affected	Related Targets	Type of Neoplasia	Description	Reference
Glycolysis	Glut1, HIF1α, Akt and mTOR	Lung carcinoma	Inhibition of (18)F-FDG intake and glycolysis, decrease in the protein level of Glut1, HIF1α, Akt, and mTOR	[186]
Glycolysis	LDH, c-Myc	Colon cancer	Suppression of glycolytic enzymes and c-Myc; increased citrate synthase—the enzyme of the Krebs cycle	[189]
Glycolysis	HK2	Non-small cell lung cancer	Reduction in EGFR, Akt and ERK1/2 activation, which impaired HK2-mediated glycolysis	[180]
Glycolysis	HK2	Hepatocellular carcinoma	Suppression of HK2 and aerobic glycolysis	[181]
Glycolysis	HIF1α	Pancreatic cancer	Molecular docking revealed resveratrol as an inhibitor of HIF1α; down-regulation of HIF1α protein level	[187]
Glycolysis	GLUT1, HK2, PFK1, PKM2	Human umbilical vein endothelial cells (HUVECs)	Suppression of VEGF-induced glycolysis; inhibition of GLUT1, HK2, PFK1 and PKM2 expression; PKM2 mislocation	[183]
Glycolysis		Ovarian cancer	Inhibition of glycolysis, activation of AMPK, and down-regulation of mTOR	[184]
Glycolysis	GLUT1 HK2 PKM2 LDHA, miR-21	Pancreatic cancer	Resveratrol decreased miR-21-mediated glycolysis	[182]
OXPHOS	ND1 ATPS ANT GA	Ovarian cancer	Inhibition of both glycolysis and respiration; decrease in ND1, ATPS, ANT, GA OXPHOS proteins	[191]
Pentose phosphate pathway (PPP)	G6PD transketolase	Colon cancer	Inhibition of PPP by down-regulation of its key enzymes—G6PD, transketolase	[188]
Metabolism of glutamine	ASCT2	Hepatoma	Suppression of glutamine importer ASCT2 enhances cisplatin sensitivity	[194]
Fatty acids biosynthesis	ACACA FASN	Breast cancer	AMPK activation, inhibition of mTOR and acetyl-CoA carboxylase α (ACACA)	[192]
Fatty acids biosynthesis	FASN	Breast cancer	Down-regulation of FASN in Her2-overexpressing breast cancer	[193]

**Figure 8:**
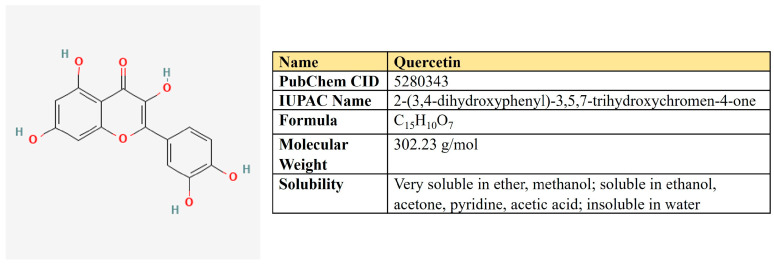
The structure and chemical properties of quercetin.

***Quercetin.*** Quercetin is a flavonoid compound (3,3′,4′,5,7-pentahydroxyflavone) that is widely distributed in different fruits and vegetables (Figure 8).

There are reports about quercetin-mediated inhibition of several key metabolic pathways in cancer cells (Table 3). First of all, quercetin directly inhibits GLUT1 (Ki = 8 µM) in acute myelogenous leukemia (AML) HL-60 cells [195].

Quercetin sensitized cells resistant to erlotinib oral squamous cell carcinoma by PKM2 inhibition. It also suppressed GLUT1, HK2, and LDHA, as well as Twist, N-cadherin, MMP-9, and MMP-13, alleviating migration, invasion, and xenograft growth [196]. In breast cancer, quercetin down-regulates Akt, induces autophagy, and suppresses glucose uptake protein levels of PKM2, GLUT1, LDHA, MMP2, MMP9, and VEGF [197].

Two research groups demonstrated that quercetin is able to bind G-quadruplex structures in the c-Myc promoter and inhibit its expression [174,198]. It was also shown that quercetin mitigates the PI3K/Akt/mTOR pathway and down-regulates c-Myc expression in Burkitt’s lymphoma [199]. Moreover, it significantly inhibited the protein level of HIF1α and sensibilized hepatocellular and pancreatic carcinoma cells to gemcitabine [200].

Not only glycolysis but OXPHOS as well was suppressed by quercetin in murine melanoma cell line [201]. Furthermore, quercetin may mitigate both fatty acids synthesis and β-oxidation. It down-regulates FASN in HepG2 cells [202] and nasopharyngeal carcinoma [203]. In the breast cancer cell model, Ruidas and colleagues have shown that quercetin down-regulates the expression level of both FASN and CPT1, as well as β-oxidation intensity and tumor growth in vivo. Moreover, the computational docking analyses predicted the binding of quercetin to CPT1 [204], which suggest the possible direct inhibitory effect of this compound on β-oxidation.

**Table 3:** Quercetin-mediated impact on metabolic pathways in cancer models.

Metabolic Pathway Affected	Related Targets	Type of Neoplasia	Description	Reference
Glycolysis	GLUT1	AML	Direct inhibition of GLUT1 (Ki = 8 µM) and glucose uptake in HL-60 cells	[195]
Glycolysis	PKM2, GLUT1, LDHA, HK2	Oral squamous cell carcinoma	Reverse of erlotinib resistance by inhibition of PKM2; decrease in invasion, migration capacities, and xenograft growth	[196]
Glycolysis	HIF1α	Pancreatic and hepatocellular carcinoma	Decrease in MDR1 activity and HIF1α protein level; increased sensitivity to gemcitabine	[200]
Glycolysis	c-Myc	Cervical and colorectal cancer	Binding of kaempferol with G-quadruplex in promotor region; decrease in c-Myc expression	[174]
Glycolysis	c-Myc	Burkitt’s lymphoma	Down-regulation of PI3K/Akt/mTOR and c-Myc	[199]
Glycolysis OXPHOS		Melanoma	Dose-dependent inhibition of both glycolysis and respiration	[201]
De novo lipogenesis	FASN	Nasopharyngeal carcinoma	Decrease in FASN and Ki-67 levels	[203]
De novo lipogenesis	FASN	Hepatocellular carcinoma	Decrease in FASN level	[202]
OXPHOS, FAO	CPT1	Breast cancer	Decreased the level of CPT1 and FASN, suppressed β-oxidation and in vivo tumor growth	[204]

**Figure 9:**
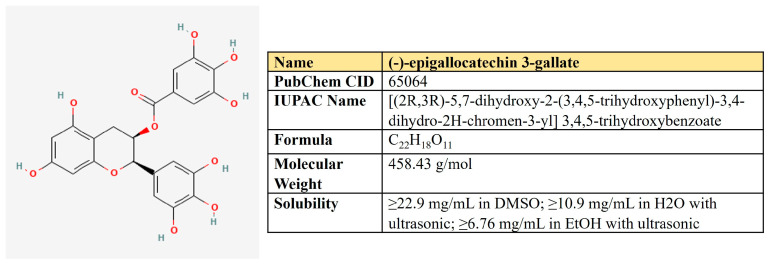
The structure and chemical properties of epigallocatechin-3-gallate.

***(−)-Epigallocatechin-3-gallate (EGCG).*** EGCG represents a polyphenolic compound (catechin), which is the ester of epigallocatechin and gallic acid (Figure 9). This is the most abundant catechin in tea. Due to its beneficial pharmacological properties, including antioxidant, cardio- and neuroprotective, antidiabetic, and cholesterol-lowering abilities, in addition to daily intake in the form of tea, EGCG is widely consumed as a dietary supplement [205]. It was shown that EGCG may prevent aging, cognitive dysfunction, and even carcinogenesis [206].

The plethora of antineoplastic properties makes EGCG a candidate for antitumor therapeutics [206] (Table 4). EGCG inhibits activation of c-Met and EGFR signaling by alteration of lipid membrane rafts [207,208] and suppresses EMT and invasion by inhibiting TGF-β1/Smad [209]. It may also alleviate STAT3, ERK NF-κB, and Akt-mediated pathways in several cancers [206]. Different studies have demonstrated that EGCG sensibilizes tumor cells to common chemotherapeutics such as doxorubicin, cisplatin, 5-FU, and tamoxifen as well as may help to reduce their adverse effects (reviewed in [210]).

Relating to cancer-associated metabolic rewiring, EGCG possesses a full spectrum of inhibitory capacities. First of all, EGCG affects glycolysis in multiple ways, demonstrating a global inhibitory effect on cell energetics. In breast cancer cells, it induced autophagy and apoptosis, decreased lactate and ATP levels, suppressed mRNA level and activity of hexokinase (HK), phosphofructokinase (PFK), and lactic dehydrogenase (LDH); decreased glucose consumption, GLUT1, and HIF1α; inhibited proliferation and xenograft growth [211]. In tongue carcinoma cells, EGCG inhibits the activation of EGFR, AKT, and ERK1/2, diminishes glucose consumption and lactate production, and decreases the protein level of HK2 and its translocation to the mitochondrial outer membrane [212].

In hepatocellular carcinoma, EGCG inhibited both the expression and activity of PFK in concentrations of 25–100 µM. It transforms the oligomeric structure of PFK into its inactive form, suppresses glucose uptake and lactate production, and induces apoptosis [213].

Using the metabolomic approach in the pancreatic cell model, Lu and colleagues have revealed EGCG-mediated perturbation of the metabolic network, down-regulation of glycolytic rate, and biosynthesis of fatty acids [214]. Finally, in colon cancer cells, EGCG interferes with membrane lipid rafts, reducing MCT1 activity, which mediates lactate export—the critical step supporting aerobic glycolysis [215].

EGCG may negatively affect the genetically altered Krebs cycle. IDH-mutant-bearing malignancies use glutamine processing to produce oncometabolite, 2-hydroxyglutarate (2-HG) [216,217]. EGCG in doses of 5–20 µM inhibited both IDH1 and GDH1/2, reduced proliferation and 2-HG production, making IDH-mutant cancer cells sensitive to irradiation [218].

Beyond the metabolism of glucose, several researchers have found EGCG as a direct inhibitor of glutamine dehydrogenase (GDH), the enzyme which [219,220]. In addition, EGCG is a direct FASN inhibitor [221,222]. Puig et al. have compared the inhibitory effects of EGCG and C75 on FASN inhibition in breast cancer cells [223]. Whereas the degree of inhibition was similar for both compounds, EGCG has a moderate inhibitory effect on fatty acids β-oxidation through a negative impact on CPT1. In contrast, C75—a commonly used FASN inhibitor—significantly stimulated CPT1. Both EGCG and C75 treatments resulted in reduced proliferation and protein levels of HER2, AKT and ERK1/2 [223]. The same results were also obtained for lung cancer [224].

In hepatocellular carcinoma HepG2 cells, EGCG simultaneously decreased FASN and ACC protein levels and reduced the activity of CPT1, which was associated with apoptosis [225].

These data demonstrate a high potential of EGCG to inhibit metabolic rewiring in cancer.

**Table 4:** EGCG-mediated impact on metabolic pathways in cancer models.

Metabolic Pathway Affected	Related Targets	Type of Neoplasia	Description	Reference
Glycolysis	HK, PFK, LDHA, GLUT1, HIF1α, VEGF	Breast cancer	Decreased glucose consumption and lactate production; induced autophagy and apoptosis; suppressed glycolytic enzymes, Glut1, HIF1α, and VEGF; inhibited xenografts	[211]
Glycolysis	HK2	Oral carcinoma	Decrease in glucose consumption and lactate production; inhibition of EGFR, AKT, and ERK activation; decrease in HK2 protein level and its translocation to mitochondrial membrane	[212]
Glycolysis	PFK	Hepatocellular carcinoma	Decrease in both PFK expression level and activity through the shift from oligomeric to inactive form	[213]
Glycolysis	MCT1	Colon cancer	Alters MCT1 membrane localization	[215]
Krebs cycle	Mutant IDH1; GDH1/2	Colorectal cancer	Inhibits IDH1 and GDH1/2; makes IDH1-mutant cells sensitive to irradiation	[218]
Glutamine metabolism	GDH	In vitro study	Directly inhibits GDH	[219,220]
Lipogenesis	FASN	In vitro study	Directly inhibits FASN	[221,222]
Lipogenesis FAO	FASN, ACC, CPT1	Hepatocellular carcinoma	Decreased FASN and ACC protein levels; reduced activity of CPT1	[225]

### 5.2. Curcumin

**Figure 10:**
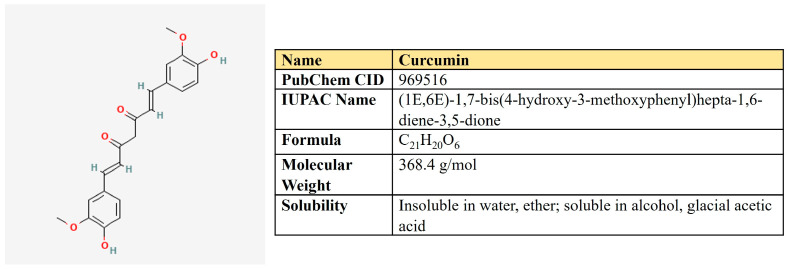
The structure and chemical properties of curcumin.

Curcumin is a polyphenolic compound that is extracted from the rhizome of turmeric (*Curcuma longa* L.) and is the main ingredient (Figure 10). Curcumin possesses a number of beneficial pharmacological properties: antioxidant, anti-inflammatory, cardio-, hepato- and neuroprotective, antidiabetic, anti-ulcer, antimicrobic, etc. [226]. It is active against breast, colorectal, gastric, prostate, and lung cancer through a variety of molecular mechanisms (Table 5). Briefly, curcumin suppresses a number of signaling pathways, including PI3K/AKT, ERK/MAPK, Wnt, and NF-kβ; inhibits proliferation, migration, and invasion; reduces stemness; and induces autophagy, ferroptosis, and apoptosis (reviewed in [226,227]).

One research group has shown that in four cancer cell lines of different origin, curcumin decreased glucose uptake, lactate production, and protein levels of HIF1α, PKM2, and p70S6K—the target of mTOR [228]. This effect was abolished upon PKM2 overexpression. Other researchers have studied curcumin-mediated hyperglycemia-induced chemoresistance in hepatocarcinoma cells. Curcumin decreased the high glucose-induced survival of cancer cells upon doxorubicin and methotrexate treatment, suppressed glucose uptake, lactate production, expression of GLUT1/3, MCT1/4, HIF1α, mTOR, STAT3, and multidrug resistance protein MDR-1 [229].

In several murine tumor models, curcumin was shown to down-regulate the activity of ATP synthase, the ATP level, and the ATP/AMP ratio both in vitro and in vivo. It also increased ROS, induced autophagy, and revealed antiangiogenic activities in B16 xenografts [230].

In MCF7 cells, the treatment with curcumin was enhanced by four times glucose uptake, lactate production, and HK activity. This was accompanied by a significant reduction in respiration 6 h post treatment as well as suppression of cell growth [231].

Taken together, it seems that the effect of curcumin on glucose uptake and the activity of glycolytic enzymes are strongly dependent on the cellular context as it can differ oppositely for cell lines of different origins. However, regardless of this, in all cases, curcumin negatively affects the growth of malignant cells.

Curcumin severely reduced the growth and migration of adrenocortical carcinoma cells and also induced apoptosis in these cells [232]. Despite the expression of some glycolytic genes being induced, both glycolysis and respiration were significantly suppressed. However, it was accompanied by enhanced expression of glutamic pyruvic transaminase (GPT), glutamine importer SLC1A5, and glutaminase (GLS1), pointing to metabolic reprogramming toward glutamine utilization. Moreover, the decrease in glutamine concentration in media significantly enhanced the cytotoxic properties of curcumin [232]. These results suggest that simultaneously targeting glutamine metabolism in line with curcumin treatment may represent a promising strategy regarding at least adrenocortical carcinoma.

In the colon cancer model, the (P-gp)-mediated multidrug resistance was closely associated with spermine and spermidine synthesis and glutamine metabolism. Curcumin suppressed these metabolic alterations, which, in turn, mitigated the antioxidant response and P-gp transport activity and eventually reversed multidrug resistance [233]. In another research, curcumin preferentially targeted colon CSCs suppressing glutamine metabolism in the CD44^+^ cell population [234]. It was shown that curcumin induces the expression of miR-137, which directly targets glutaminase mRNA [235]. This attenuated glutamine metabolism and sensitized colorectal cancer cells to cisplatin.

Beyond glucose and glutamine metabolism, curcumin possesses a high potential to target the metabolism of lipids, including lipogenic enzymes FASN, ACC, and ACLY, as well as their transcriptional regulators, SREBP1 [236]. Thus, curcumin down-regulated both the expression level and enzymatic activity of FASN in hepatocellular (HCC) [237] and breast [238,239] carcinoma. In HCC murine model, curcumin significantly enhanced sorafenib activity, increased the amount of CD4^+^ T-cells and NK-cells, down-regulated p-PI3K/p-Akt, HIF1α, FASN, SREBP1, and CPT1a. Moreover, the computer modeling proposed the potential binding of curcumin with FASN, STAT3, and AKT [240].

In addition, a wealth of data on curcumin down-regulating lipid metabolism comes from studies on adipocytes, hepatics, and other non-tumor cells and tissues. For instance, curcumin suppressed genes involved in cholesterol biosynthesis, FASN, ACC, SREBP1, and PPARγ [236].

In another study, Yang and colleagues developed nanoparticles carrying curcumin, which efficiently targeted both PKM2 and FASN and attenuated energy metabolism in breast cancer cell models [241].

**Table 5:** Curcumin-mediated impact on metabolic pathways in cancer models.

Metabolic Pathway Affected	Related Targets	Type of Neoplasia	Description	Reference
Glycolysis	HIF1α PKM2	Cancer cell lines of different origin	Decreased glucose uptake, lactate production, and protein levels of HIF1α and PKM2	[228]
Glycolysis	GLUT1/3MCT1/4 HIF1α	Hepatocellular carcinoma	Suppressed glucose uptake, lactate production, expression of GLUT1/3, MCT1/4, HIF1α, mTOR, STAT3, and multidrug resistance protein MDR-1	[229]
OXPHOS	ATP synthase	Breast cancer	Suppressed the activity of ATP synthase, ATP level, and ATP/AMP ratio both in vitro and in vivo	[230]
OXPHOS		Breast cancer	Increased glucose uptake, lactate production, and HK activity but suppressed respiration and cell growth	[231]
Metabolism of glutamine	GLS1	Colon cancer	Induces miR-137 expression, which directly targets GLS1	[235]
De novo lipogenesis	FASN	Hepatocellular carcinoma	Down-regulated both expression level and enzymatic activity of FASN	[237]
De novo lipogenesis	FASN	Breast cancer	Down-regulated both expression level and enzymatic activity of FASN	[238]
De novo lipogenesis FAO	FASN SREBP1 CPT1a	Hepatocellular carcinoma	Down-regulated p-PI3K/p-Akt, HIF1α, FASN, SREBP1 and CPT1α in murine cancer model	[240]

**Figure 11:**
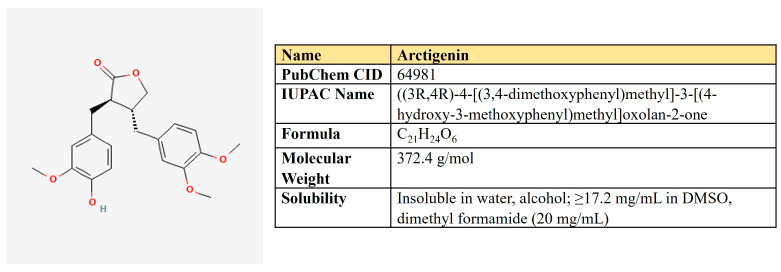
The structure and chemical properties of arctigenin.

***Arctigenin.*** Arctigenin (Arc) is a lignan (Figure 11) that is found in *Arctium lappa,* which possesses antioxidant, anti-inflammatory, antiviral, and anticancer activities [242]. A broad spectrum of antineoplastic properties was shown for this compound in various neoplasia [243].

In different cancer models, Arc suppressed EGFR- and Her2-mediated signaling cascades [244,245], Akt/mTOR- [246], and STAT3/β-catenin-dependent pathways [247,248]. It induces cell cycle arrest, apoptosis, and autophagy, suppresses metastasis and angiogenesis, and sensibilizes malignant cells to chemotherapeutics [249,250].

In contrast to other compounds reviewed, Arc does not inhibit glycolysis. However, it suppressed OXPHOS and lipid metabolism (Table 6). In the lung cancer cell model, Arc inhibited mitochondrial respiration and ATP production. It also synergized with 2-DG to induce preferential cell death of cancer but not normal cells [251]. The inhibitory effect of Arc on respiration was also shown for pancreatic cancer. Brecht and colleagues revealed that, mechanistically, Arc targets mitochondrial chain complexes II and IV and selectively kills only the OXPHOS-dependent pancreatic cancer cells [252]. It should be noticed that Arc targets respiratory chain complex I in skeletal muscles, which induces AMPK activation and has beneficial effects on metabolic disorders in obese mice models [253].

Brecht and colleagues have shown that Arc-mediated targeting of the OXPHOS-dependent pancreatic cancer cells was accompanied by ER stress induction (increase in GRP78, CHOP, and ATF4) [252]. However, under glucose deprivation conditions, Arc was able to suppress the unfolded protein response by GRP78, GRP94, and ATF4 decrease [256].

Different types of antineoplastic therapy induce ER stress. Unfolded protein response mediators, such as GRP78, PERC, CHOP, ATF4, and others, are responsible for ER stress mitigation, which leads to the survival of malignant cells. Thus, targeting ER stress proteins is now considered an antineoplastic approach [257,258].

Collectively, these data suggest the potential use of Arc in combination with any compounds targeting glycolysis and glucose uptake. The glucose deprivation and inhibition of glycolysis induce both ER stress and respiration as an adaptation way to prevent deficiency of energy [259,260]. As Arc suppresses respiration and ER stress upon glucose deficiency, it may confer a synergistic effect with inhibitors of glucose uptake and glycolysis.

Arc may affect not only respiration but also has inhibitory activity toward the metabolism of fatty acids. NLRP3 inflammasome plays an important role in the development of colitis and colorectal cancer [261]. Qiao and colleagues have established a mouse model of induced colorectal cancer and studied the potential therapeutic effect of Arc [254]. They observed Arc-mediated down-regulation of NLRP3 inflammasome activity and β-oxidation of fatty acids in macrophages. Mechanistically, the metabolomic and metabolic assays revealed that Arc decreased FAO, suppressed expression level, and enzymatic activity of CPT1 [254]. Thus, Arc prevented the progression of colitis and protected against colon carcinogenesis.

In another study, Arc was shown to down-regulate peroxisome proliferator-activated receptor-gamma (PPARγ) and CCAAT/enhancer-binding protein-alpha (C/EBPα) in differentiated adipocytes, which also implies the inhibitory activity of this natural compound on fatty acid metabolism [255].

A number of studies reported the negative impact of Arc on the Akt/mTOR pathway, which boosts anabolic processes in malignant cells. Arc down-regulates Akt and mTOR phosphorylation and induces autophagy in prostate, breast, hepatic cancer, and glioblastoma [248,262,263,264].

### 5.3. Shikonin

**Figure 12:**
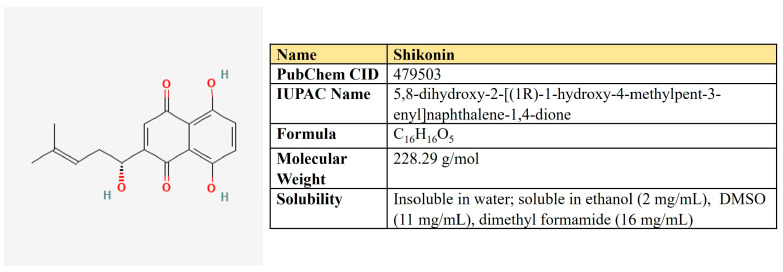
The structure and chemical properties of shikonin.

Shikonin, a naphthoquinone compound extracted from the root of *Lithospermum erythrorhizon* (Figure 12).

In malignant cells, shikonin suppressed ERK and β-catenin-mediated signaling [265], up-regulates p21 and arrests cells in G2/M [266], targets cell division cycle 25 (Cdc25) phosphatases [267], induces apoptosis via activation of FOXO3a/EGR1/SIRT1 [268]. Also, it has a negative impact on cancer metabolism (Table 7).

In lung cancer cells, shikonin suppressed PFKB at both mRNA and protein levels. It down-regulated proliferation, migration, invasion, glucose uptake, ATP, and lactate production in doses of 10–50 µM, as well as increased the number of apoptotic cells [269].

Chen et al. have studied the inhibitory activity of shikonin on a set of glycolytic enzymes in the cell extract derived from MCF7 breast cancer cells [270]. The authors carried out a 1 h incubation of cells with shikonin followed by the measurement of enzymatic activity. Interestingly, the concentration of shikonin inside cells was higher than its extracellular concentration, which means that cells may accumulate shikonin. IC50 values for enzyme inhibition were 9.7, 17.2, 96.8, 89.5, and 12.2 µM for HK, PFK-1, PGI, PGK, and PK, respectively [270].

However, first of all, shikonin is known as a PKM2 inhibitor. Shikonin was shown to significantly inhibit in vitro and in vivo growth of tumor cells in orthotopic mice models—Lewis carcinoma and B16 melanoma [271]. In these systems, shikonin ameliorated PKM2 phosphorylation but did not alter its protein level. Shikonin reduced glucose uptake, and lactate production induced apoptosis in doses of 10–20 µM [271].

In esophageal squamous cell carcinoma patient-derived xenografts, shikonin down-regulated p-PKM2, HK, GLUT1, and p-STAT3, glucose uptake, and lactate production, which was accompanied by suppression of tumor growth [272].

Cisplatin-based chemotherapy is often overcome in bladder cancer. However, shikonin was reported to be able to overcome cisplatin resistance, which was dependent on the inhibitory effect on PKM2 and aerobic glycolysis [273].

In addition, shikonin prevents di- and tetramerization of PKM2 in macrophages, which ameliorates colitis in mice [274].

Noteworthy, shikonin suppressed the growth of cholangiocarcinoma cells and induced apoptosis of up to 70% in a dose of 0.5–1.5 µM, which means that these types of tumors may be extremely susceptible to this compound [275]. In colorectal cancer, shikonin inhibited HIF-1α protein synthesis without affecting the expression of HIF-1α mRNA or degrading HIF-1α protein, which leads to inactivation of mTOR/p70S6K/4E-BP1/eIF4E [276].

Beyond the energy metabolism, shikonin is reported to suppress the important enzyme of amino acid metabolism—PYCR1, in T cell leukemia/lymphoma, which, in pair with ALDH18A1, makes proline from glutamate. This was associated with up-regulation of autophagy and apoptosis [277]. It is noteworthy that PYCR1, together with ALDH18A1, are the two most overexpressed enzymes among 19 tumor types and the most up-regulated genes in hepatocellular carcinoma [278].

Chen and colleagues have also shown that even 1 µM of shikonin was sufficient to down-regulate more than 50% of colon carcinoma cells. The authors have coupled transcriptomic and metabolomic data and demonstrated that purine and pyrimidine metabolism, as well as arginine biosynthesis and metabolism of other amino acids, were affected by shikonin intervention. Furthermore, supplemental dNTPs and arginine rescued shikonin-induced cytotoxicity [279]. These results suggest a global negative impact of shikonin on cancer metabolic networks.

**Table 7:** Shikonin-mediated impact on metabolic pathways in cancer models.

Metabolic Pathway Affected	Related Targets	Type of Neoplasia	Description	Reference
Glycolysis	PFK	Lung cancer	Down-regulated PFK, ATP, and lactate production	[269]
Glycolysis	HK, PFK, PGI, PGK, PK	Breast cancer	Down-regulated a number of glycolytic enzymes	[279]
Glycolysis	PKM2	Lung cancer and melanoma mouse models	Inhibited PKM2 phosphorylation, glucose uptake, and lactate production	[271]
Glycolysis	GLUT1, PKM2, HK	Esophageal squamous cell carcinoma	Reduced the number of glycolytic enzymes, glucose uptake, and lactate production	[272]
Glycolysis	HIF-1α	Colorectal cancer	Inhibited HIF-1α protein synthesis; inactivated mTOR/p70S6K/4E-BP1/eIF4E axis	[276]
Amino acid metabolism	PYCR1	T cell leukemia/lymphoma	Down-regulated PYCR1, which is involved in proline synthesis	[277]
Biosynthesis of nucleotides and amino acids		Colon carcinoma	Interfered with purine and pyrimidine metabolism, as well as arginine biosynthesis	[279]

### 5.4. Betulinic Acid

**Figure 13:**
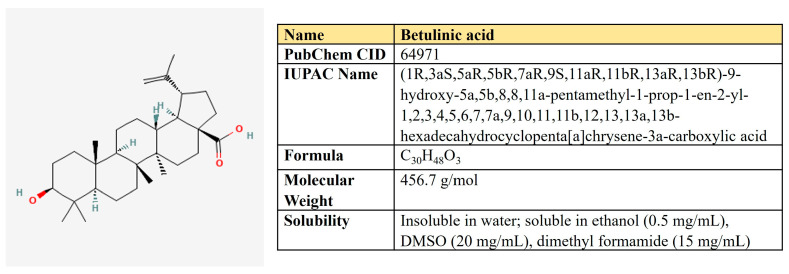
The structure and chemical properties of betulinic acid.

Betulinic acid is a pentacyclic triterpene of the lupane type derived from birch bark extracts (Figure 13). Dozens of studies have demonstrated the potential of BA to treat malignancies and immunological disorders [280,281]. In cancer cells, BA induced ROS production and autophagy, activated the mitochondrial apoptotic pathway, and inhibited EMT [282].

BA hampered the intensity of glycolysis, glucose uptake, and lactate production and suppressed c-Myc, LDHA, and PDK1 in breast cancer cells [283] (Table 8). In human melanoma cells, BA decreased both glycolysis and respiration in a dose-dependent manner [284]. Regarding breast cancer, BA also suppresses glycolysis and the development of metastasis in vivo. Mechanistically, it prevents the interaction of glucose-regulated protein 78 (GRP78) with the endoplasmic reticulum stress sensor (PERP), which leads to the inhibition of β-catenin and c-Myc expression, as well as c-Myc-mediated glycolysis [285]. In lung cancer cells, BA also down-regulated c-Myc and cancer stem cell markers CD133 and ALDH [286]. In the other study, BA suppressed aldolase, enolase, LDHA, and PKM2 in colorectal cancer [287].

Besides glycolysis, BA is shown to negatively regulate the metabolism of glutamine and lipids. In recent studies, BA-loaded liposomes efficiently targeted glycolysis, glutaminolysis, and fatty acid metabolism. In the research of Wang and colleagues [287], such liposomes suppressed proliferation and glucose uptake, decreased glycolytic enzymes HK2, PFK-1, PEP, and PKM2 as well as an important enzyme of fatty acids biosynthesis—ACSL1, and the rate-limiting FAO enzyme—CPT1a.

It was also shown that BA directly inhibits glutaminase with IC50 of 0.31 mM, although this concentration seems rather high [288].

Although no information is available for malignant cells yet, a couple of papers describe that BA lowers lipid accumulation in adipocytes by modulating PPARγ [289,290]. However, the potential impact of BA on lipid metabolism in cancer remains to be addressed.

**Table 8:** Betulinic acid-mediated impact on metabolic pathways in cancer models.

Metabolic Pathway Affected	Related Targets	Type of Neoplasia	Description	Reference
Glycolysis	c-Myc LDHA	Breast cancer	Decrease in c-Myc, LDHA, and PDK1; down-regulation of glycolysis	[283]
Glycolysis OXPHOS		Melanoma	Dose-dependent down-regulation of glycolysis and respiration	[284]
Glycolysis	GRP78, β-catenin, c-Myc	Breast cancer	Interferes with GRP78/PERP interaction, decreases β-catenin and c-Myc expression, suppresses glycolysis	[285]
Glycolysis	HK2 PFK-1 PKM2 ACSL1 CPT1A	Colorectal cancer	Liposome-loaded BA decreased proliferation, glucose uptake, suppressed enzymes indicated	[291]
Glutaminolysis	GLS	In vitro study	Direct inhibition of GLS (IC50 = 0.31 mM)	[288]
Glycolysis	c-Myc	Lung cancer	Suppressed c-Myc, cancer stem markers ALDH, CD133, and anti-apoptotic proteins Bcl2 and Mcl1	[286]

### 5.5. Cucurbitacins

**Figure 14:**
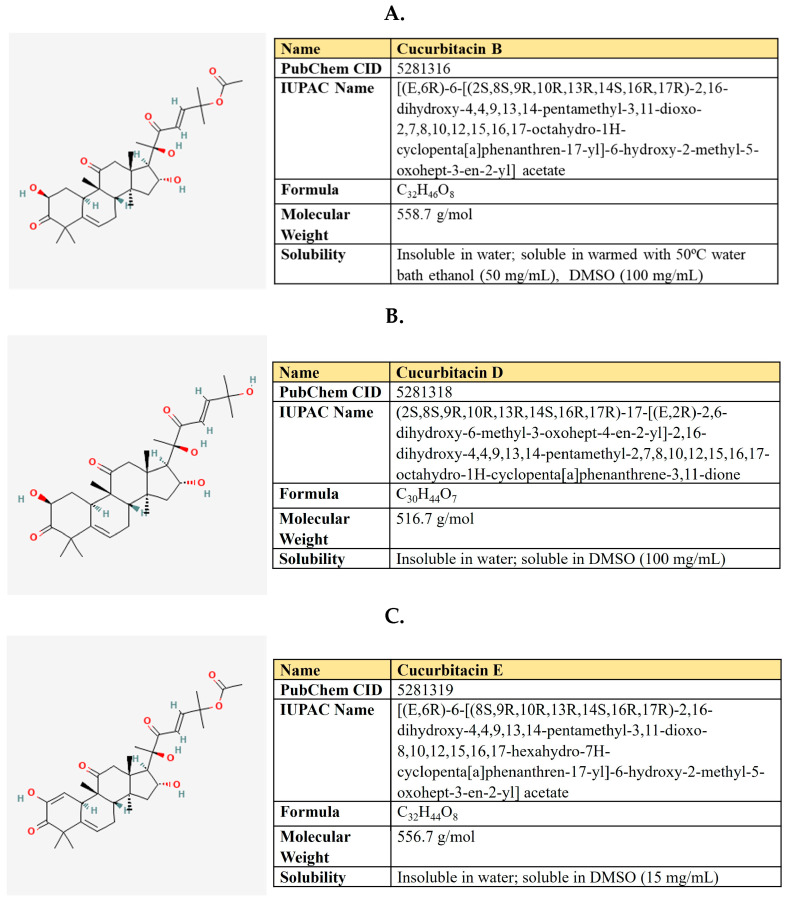
The structure and chemical properties of cucurbitacin B (**A**), cucurbitacin D (**B**), and Cucurbitacin E (**C**).

Cucurbitacins are groups of tetracyclic triterpenoid compounds produced by members of the *Cucurbitaceae* family, including cucumber, pumpkin, melon, and watermelon (Figure 14). These compounds attract attention by their anticancer, anti-inflammatory, antiviral, antimicrobial, hyperglycemic, antioxidant, and hepatoprotective properties in humans (reviewed in [292]). Cucurbitacin B (CucB) has been used in Chinese medicine in the form of tablets.

According to the literature data, they confer strong antineoplastic effects regarding different types of malignancies—breast, lung, prostate, pancreatic, gastric, etc. [293], negatively affecting Jack/STAT, NFκB, PI3K/Akt/mTOR, MAPK/ERK, and Wnt/β-catenin signaling pathways [292]. A number of studies have shown cucurbitacin-mediated sensitization of neoplastic cells to anticancer therapeutics, including doxorubicin, paclitaxel, dodetaxel, cisplatin, irinotecan, gemcitabine, and methotrexate (summarized in [294]).

In prostate cancer, cucurbitacin D in sub-micromolar concentration decreased glucose uptake and lactate production and reduced AKT, GLUT1, and the c-Myc protein level (Table 9). It mediated G2/M cell cycle arrest and apoptosis and attenuated the growth of xenografts [295]. In another research on prostate cancer, cucurbitacin B also reduced xenograft growth and induced apoptosis, which was dependent on CucB-mediated down-regulation of ACLY phosphorylation [296]. Ji and colleagues investigated the impact of CucB on tumor metabolism of c-Met/AKT-overexpressing hepatocellular carcinoma in mice. They observed the inhibitory activity of CucB on several metabolic networks, including de novo lipogenesis. CucB suppressed the activity of AKT and mTOR, glycolytic enzymes HK2 and PKM2, down-regulated SPEBP1, and its two main transcriptional targets—FASN and ACC [297].

In breast cancer cells, CucB inhibited the expression of telomerase (hTERT) and c-Myc [298]. Interestingly, in other research on breast cancer, the authors have reported that CucB significantly elevated the level of DNMT1 and induced extensive methylation of the promoter regions of c-Myc, cyclin D1, and survivin genes, resulting in the down-regulation of these oncogenes and suppression of the growth of cancer cells [299].

**Table 9:** Cucurbitacins-mediated impact on metabolic pathways in cancer models.

Metabolic Pathway Affected	Related Targets	Type of Neoplasia	Description	Reference
Glycolysis	GLUT1 c-Myc	Prostate cancer	0.1–1 µM CucD inhibited glucose consumption and lactate production	[295]
De novo lipogenesis	ACLY	Prostate cancer	0.3 µM CucB inhibited xenograft growth in an ACLY-dependent manner	[296]
Glycolysis De novo lipogenesis	mTOR HK2 PKM2 SPEBP1 FASN ACC	Hepatocellular carcinoma	CucB down-regulates a number of metabolic enzymes in AKT/c-Met-induced HCC mice	[297]
Glycolysis	c-Myc cyclin D1	Breast cancer	CucB induced DNMT1-mediated methylation of c-Myc and cyclin D1 promoters	[299]
Glycolysis	c-Myc	Laryngeal carcinoma stem cells (LCSCs)	CucE decreased c-Myc, ABCG2, and P-gp in LCSCs	[300]
Glycolysis	HIF-1α	Cervical carcinoma	CucB inhibited HIF-1α protein synthesis without any impact on the transcriptional level both in vitro and in vivo	[301]

The treatment of osteosarcoma cell lines with cucurbitacin I inhibited STAT3 signaling and decreased the expression of cyclin D1, Mcl-1, c-Myc, and survival [302].

Jiang and colleagues have studied the influence of CucE on the properties of laryngeal carcinoma stem cells (LCSCs) [300]. The authors have shown that treatment with CucE significantly decreased the invasion potential of LCSCs and suppressed xenograft growth a quarter more effectively than doxorubicin, as well as enhanced doxorubicin-mediated toxicity. Moreover, CucE decreased the protein level of c-Myc and multidrug resistance proteins ABCG2 and P-gp, which are referred to as LCSC markers [300]. Besides c-Myc, CucB was shown to inhibit HIF-1α protein synthesis without any impact on the transcriptional level in cervical carcinoma cells both in vitro and in vivo. This effect was accompanied by the inactivation of ERK1/2, mTOR, and its down-stream effectors: ribosomal protein S6 kinase (p70S6K) and eukaryotic initiation factor 4E-binding protein-1 (4E-BP1) [301].

In non-small cell lung adenocarcinoma cells, cucurbitacin B induced the lysosomal degradation of EGFR in a dose of 0.01–0.1 µM and strongly suppressed xenograft growth. This effect is of high translational value because mutant EGFR confers constitutive proliferation to cancer cells and is resistant to standard anti-EGFR therapies, creating problems for lung cancer therapy. By being active at the level of EGFR lysosomal degradation, CucB may effectively target cancer cells bearing either wild-type or mutant EGFR [303].

### 5.6. Ginsenosides

**Figure 15:**
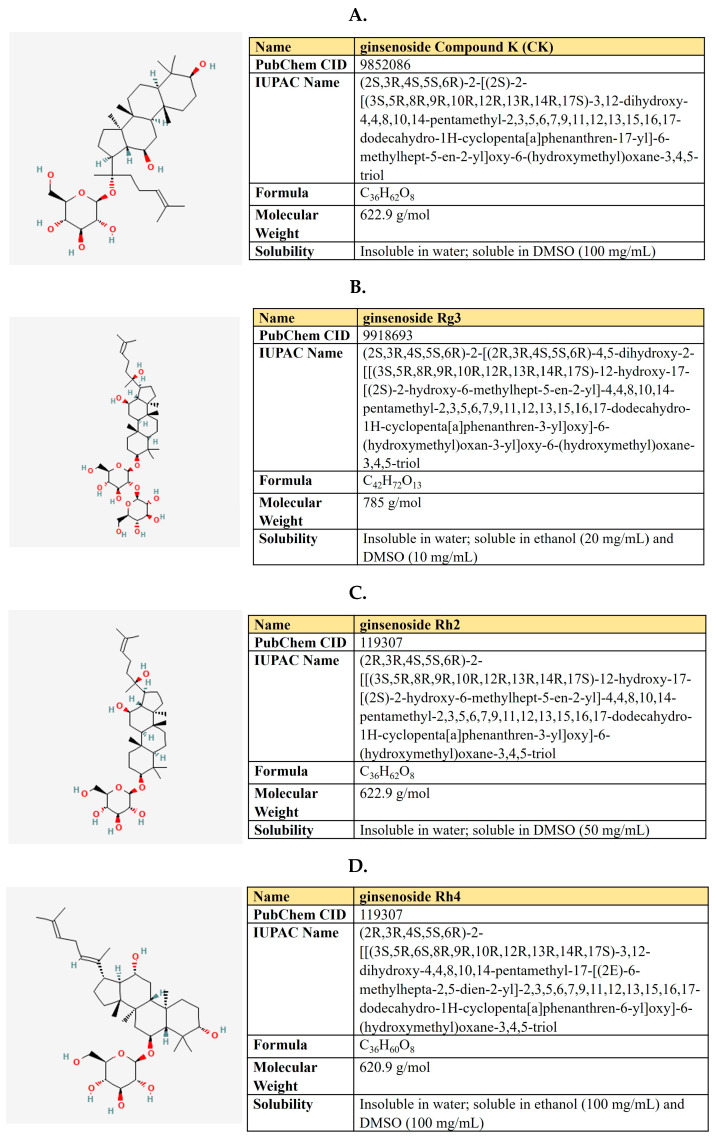
The structure and chemical properties of ginsenosides: compound K (**A**), Rg3 (**B**), Rh2 E (**C**), and Rh4 (**D**).

Ginsenosides is a group of compounds that are principally responsible for the medical attributes of ginseng—one of the most popular plants of traditional medicine. Regarding chemical structure, ginsenosides are steroidal saponins with a triterpenoid dammarane nature, having a four-ring, steroid-like configuration with sugar moieties conjugated (Figure 15). About 100 different ginsenosides have been isolated from roots, stems, and leaves of *Panax* species, and notably, 10 of them are characterized and possess well-known pharmacological properties [304,305].

Dozens of studies have been published that reveal ginsenoside’s potential to treat diabetes [306], inflammatory [307], and neurodegenerative [308] diseases and cancer [305]. Ginsenosides exercised antineoplastic properties in multiple ways, including inactivation of EGFR, Akt, ERK, and STAT3 signaling, inhibition of EMT markers, migration, invasion, and angiogenesis. Also, they can reverse multidrug resistance induced by MDR1 and MRP1 and induce autophagy, ROS, and apoptosis (reviewed in [309,310]).

Different ginsenosides target metabolic rewiring in neoplastic cells [310] (Table 10). A number of studies reported about ginsenosides-mediated down-regulation of PI3K/AKT/mTOR pathway and glycolysis. In a rat model, Rg3 mitigates gastric precancerous lesions by reducing angiogenesis. Mechanistically, it down-regulated VEGF, GLUT1, and GLUT4 in both the rat model and human gastric cancer cells [311]. In hepatocellular carcinoma cells, Rg3 significantly enhanced the activity of sorafenib. The Rg3/sorafenib combinational treatment attenuates p-PI3K, p-Akt, and HK2 [312].

In esophageal carcinoma, ginsenoside Rh4 in a dose of 20–60 µM was able to decrease the protein level of GLUT1, HK2, PFKL, PKM2, LDHA, p-Akt, and p-mTOR. This observation was associated with a decrease in both glycolysis and respiration levels, ATP, and lactate production [313]. In NSCLC, ginsenoside Rh2 targeted the STAT3/c-Myc axis, which resulted in GLUT1, PKM2, and LDHA inhibition and was associated with EMT suppression and apoptosis induction [314].

Another ginsenoside, compound K (CK), attenuated AKT/mTOR/c-Myc signaling in hepatocellular carcinoma, which also led to HK2 and PKM2 suppression and apoptosis [315].

Besides the AKT/mTOR/c-Myc axis, ginsenosides suppressed HIF1α-dependent pathways. In hypoxic hepatoma cells and xenografted models, ginsenoside CK in a dose of 20–60 µM down-regulated the protein level of HIF1α, glycolysis, GLUT1, glycolytic enzymes HK2, and LDHA, as well as PDK1 [316]. In ovarian cancer, ginsenoside Rh3 up-regulates miR-519a-5p, which, in turn, targets HIF1α. It inhibits DNMT3A-mediated DNA methylation in the promoter region of miR-519a-5p [317].

Ginsenoside Rk1 suppressed ERK/c-Myc signaling, down-regulated glutaminase GLS1, and decreased glutathione production, which stimulates ROS and apoptosis in hepatocellular carcinoma [318].

Another report about ginsenoside’s impact on glutamine metabolism comes from triple-negative breast cancer. Zhang and co-authors have reported that the treatment with ginsenoside CK down-regulates glutamine transporter ASCT2, glutaminase GLS1, glutamine dehydrogenase GLUD1 (GDH), and their transcriptional regulator c-Myc at both mRNA and protein levels [319]. In line with the glutamine uptake and glutaminolysis suppression, CK increased the glutamine level and decreased glutamate, proline, aspartate, and asparagine. Moreover, CK inhibited ATP and glutathione production, hence increasing ROS [319].

There is also evidence about ginsenosides-mediated potential negative impact on lipid metabolism. Thus, ginsenosides Rb1, Rg1, Rg3, and CK reduce intracellular cholesterol and promote cholesterol efflux in glioblastoma cells, which interferes with lipid rafts distribution on membranes and reverses temozolomide resistance [320]. Regarding lipid metabolism in non-cancer models, ginsenosides are known to suppress FASN and SREBP1 [321,322].

**Figure 16:**
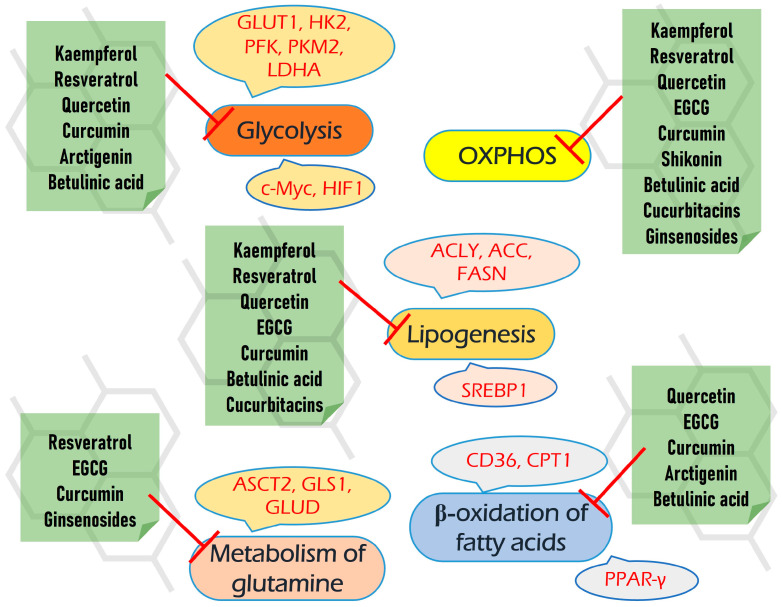
The summarizing diagram of how plant-based compounds impact different biochemical pathways (explanations are provided in the text).

Thus far, we have discussed several plant-derived natural compounds that negatively affect various metabolic pathways in malignancies. The summarizing diagram of how these compounds impact different biochemical pathways is shown in Figure 16.

## 6. The Bioavailability and Safety of Compounds Reviewed

Despite the prominent antineoplastic properties of natural compounds, their translation to human studies and therapy is frequently limited due to their low bioavailability. Bioavailability is the fraction of an ingested substance that is absorbed by the body and is available for participation in physiological activities. It is derived from absorption, distribution, metabolism, and excretion (ADME). Low water solubility is one of the main challenges. Metabolism of compounds includes mainly intestine (by both microbiota and enterocytes) and liver metabolism. Generally, there are phase I and phase II drug-metabolizing enzymes, including cytochrome P (CYPs) and UDP-glucuronosyltransferases (UGTs) [7,323].

Normally, the bioavailability and bioactivity of a certain compound may be improved by the use of either bioenhancers or drug carrier systems, which may include liposomes, silver or silica nanoparticles, PLGA (poly-lactic-co-glycolic acid), PLA (poly(D,L-lactic acid)) nanoparticles, polymeric micelles, chitosan nanoparticles, and other types [324]. For instance, self-microemulsifying drug delivery systems (SMEDDSs) are frequently developed, which are isotropic mixtures of oils, surfactants, or (alternatively) co-surfactants and co-solvents. The application of SMEDDSs significantly improved the stability, effectiveness, peak drug concentration (Cmax), and area under the curve (AUC) values of curcumin, quercetin, and resveratrol [325].

At least one clinical trial with control phase III significant results is required for the Food and Drug Administration (FDA) and the Europe Medicine Agency (EMA) to launch the compound into clinical use [7,326]. Even the widely consumed substances may display a hazardous toxicity level when they are ingested in increased doses. For example, serious hepatotoxicity occurred in people who consumed excessive amounts of green tea or its extracts as dietary supplements.

Thus, to translate into therapeutics, all-natural compounds should be studied in a panel of preclinical and clinical trials regarding their safety. Below, we briefly discuss the bioavailability and safety properties of the natural compounds reviewed.

***Kaempferol.*** Kaempferol has low water solubility, bioavailability, and absorption [164]. Thus, the intake of 15 mg of kaempferol in humans resulted in a plasma concentration of 58 nM [327]. However, experiments in rats revealed an extremely short half-life of kaempferol, which was about 4 min [328].

To address the poor bioavailability, kaempferol-carrying nanoparticles were developed, which have both in vitro and in vivo increased antineoplastic activity toward hepatocellular carcinoma [329,330].

***Quercetin.*** Although a number of studies have shown a direct link between ingested quercetin and its beneficial biological activities in humans (summarized in [331]), its bioavailability is often limited by poor water solubility and chemical stability in foods and the human gut [332]. Thus, different encapsulation technologies have been applied to improve quercetin bioavailability (reviewed in [333,334]). For instance, recently, quercetin-loaded PLGA nanoparticles were developed which efficiently targeted mammary adenocarcinoma in rats [335].

There are four clinical studies on quercetin safety (reviewed in [331]). In all of them, quercetin was ingested as aglycone; a single dose ranged from 150 to 5000 mg. Only a small increase in TNF-α was reported in one study [336], whereas other studies have reported no significant side effects [331]. Thus, quercetin has an FDA (Food and Drug Administration) status “Generally Recognized as Safe (GRAS)” as a food supplement up to 500 mg per serving.

***EGCG.*** Despite its hydrophilic nature, EGCG has a low oral bioavailability, which is the lowest among other catechins. It has extremely low stability post digestion, with <10% available for absorption [337]. Interestingly, the use of green tea with milk, ascorbic acid, or juices significantly ameliorates EGCG bioavailability. Besides low stability, metabolism, and biotransformation, which occur in the mouth, intestine, and liver, further decrease bioavailability [338]. Despite this, several studies have shown that taking the decaffeinated green tea extracts in capsules—Polyphenon E, Teavigo^®^, and FontUp^®^, which represent a catechin mixture on an empty stomach after an overnight fast may enhance bioavailability and lead to its significant blood concentrations [339,340].

However, nano-delivery systems that significantly improve EGCG bioavailability and bioactivity have been developed, including different types of nanoparticles, nanoemulsions, and nanoliposomes [210,341].

Several clinical studies confirmed the safety of EGCG [342,343]. However, high doses (more than 800 mg) can be associated with hepatotoxicity [344,345,346]. The daily intake of doses equal to or above 800 mg EGCG may be associated with a significant increase in serum transaminases [347], whereas up to 704 mg EGCG/day consumed in beverage form or 338 mg EGCG/day ingested as a concentrated solid bolus dose is considered as safe [348].

***Resveratrol.*** Resveratrol exists in two geometrical isomers—the trans- and -cis forms. The cis- form arises from the -trans one by isomerization under UV light and high pH [349]. Whereas generally, the -trans form of resveratrol is sought to be more biologically active, the -cis form may also have beneficial properties that are not similar to the -trans isoform [350].

Despite about 70% of resveratrol absorption and a peak plasma concentration of 2 µM after administration of 25 mg, it has low bioavailability due to extensive metabolism in both the intestine and liver, which results in sulfate and glucuronic acid conjugation and hydrogenation of the aliphatic double bond [351,352]. The dose escalation up to 5 g led to the increase in unchanged resveratrol up to 530 ng/mL [353]. In order to increase its bioavailability, a couple of dozen resveratrol nanoformulations have been developed, which efficiently suppress tumor growth [354,355].

A number of clinical studies have provided evidence about the safety of resveratrol [349,356,357]. Randomized clinical trials have shown that a daily intake of 500 mg resveratrol was safe and improved body mass index and insulin secretion in patients with diabetes [358,359]. Generally, resveratrol is well tolerated at doses of up to 5 g/day; however, mild to moderate side effects may occur at a dose of more than 1 g/kg [357]. Several clinical studies (summarized in [179]) suggest that patients with colon, gastric, and hepatic cancer may benefit from the administration of resveratrol.

***Curcumin.*** As the major component of turmeric, curcumin has been used by people for several millennia and is called the “wonder drug of life”. According to the Food and Drug Administration (FDA) classification, turmeric is Generally Recognized as Safe (GRAS), and the consumption of curcumin at a dose of 3 mg/kg body weight is permitted. Due to its high biological activity and suitable safety, curcumin is now free-marketed as a food supplement. It has been successfully studied in a dozen clinical trials (reviewed in [360]) to treat various diseases, including cancers [361].

The disadvantage of curcumin is that it has a low bioavailability which is collectively raised from low water solubility, limited gastrointestinal tract absorption, quick metabolism, rapid systemic clearance, and restricted blood–brain barrier penetration [226].

To improve bioavailability, a number of adjuvants (EGCG, piperine) and curcumin nanoformulations (nanoparticles, nanoemulsions, nanocomposite, hydrogels) were developed, including patented and commercially available ones (reviewed in [226,362]). For instance, curcumin in the form of galactomannoside complex (CurQfen^®^) has a significantly improved bioavailability, blood–brain barrier permeability, and cellular uptake and demonstrates safety in clinical trials in a dose of ~380 mg of curcuminoids consumed by healthy volunteers for 90 days [363,364].

Taking together high biological activity and suitable safety, it seems that curcumin has big potential as an adjuvant in antineoplastic therapy.

***Shikonin.*** The intake of 200, 400, and 800 mg/kg shikonin for several months by Wistar rats did not elicit any toxicity [365]. In addition, none, or minor, adverse effects were observed on Beagle dogs ingested 100–2000 mg/kg shikonin during 1–3 weeks [366]. Moreover, shikonin has been used in six clinical trials (summarized in [367]), half of which were aimed at treating cancer and leiomyoma. However, shikonin is reported to be a reversible inhibitor of UGT (UDP-glucuronosyltransferases) and may potentially display toxicity in drug–drug or food–drug interactions [368]. It may also inhibit members of the cytochrome P450 family [369]. Generally, in vitro toxicity of shikonin is much higher than in vivo [367], which may be attributed to its low bioavailability.

A number of nanodrug-carrying systems were developed to improve it. Albreht and colleagues have shown that the solubility of shikonin can be increased up to 181-fold by the addition of β-lactoglobulin [370]. On the other hand, the encapsulation of shikonin in nanoparticles coated with saponin and sophorolipid significantly improved its solubility and bioavailability [371]. In another research, shikonin was loaded to MPEG-PCL micelles (methoxy poly (ethylene glycol)-b-poly (ϵ-caprolactone)) which effectively inhibited EMT in endothelial model cells [372].

Furthermore, shikonin encapsulated in liposomes has displayed increased activity in vivo (reviewed in [373]). Thus, hyaluronic acid-coated shikonin liposomes were prepared for efficient targeting of TNBC (triple-negative breast cancer) cells through CD44-mediated endocytosis [374]. In another study, membrane-camouflaged micelle loaded with shikonin were developed to target TNBC tumors in mice [375].

***Arctigenin.*** Arctigenin has a poor bioavailability and an extensive first-pass metabolism [376]. The oral administration of 70 mg/kg arctigenin to rats led to its tissue concentration peaking at 30 min and was quickly eliminated within 4 h. The highest concentration of arctigenin was observed in the spleen, followed by the liver and other organs [377]. In a clinical trial involving pancreatic cancer patients, the oral dose of *Arctium lappa* extract GBS-01 at a dose of 12 g arctigenin per person, its peak concentration in the plasma was 66.56 ± 26.81 ng/mL, with AUC487.97 ± 368.86 ng·h/mL [378].

The absorption, distribution, metabolism, and elimination of arctigenin in in vitro and in vivo models and clinical trials are summarized in [376].

The safety of arctigenin remains not well studied yet. One work reports that it may be toxic for breast non-tumor cells [379]. However, a phase I clinical trial of an arctigenin-rich burdock fruit extract (GBS-01) was performed in 15 patients with advanced pancreatic cancer refractory to gemcitabine. The authors reported that the dose of 4 g/day of GBS-01 resulted in favorable clinical responses and no significant toxicity [378]. Moreover, arctigenin was also studied in three other clinical trials, revealing its efficiency in treating diabetic nephropathy [376].

***Cucurbitacins***. Cucurbitacin B is the most studied compound among all cucurbitacins, and hence, the literature information about the bioavailability and safety of this compound is the most comprehensive. Experiments with Wistar rats have shown that the oral bioavailability of cucurbitacin B was 10%, with the highest concentration in plasma ranging from 1 to 100 ng/mL and reaching maximum value approximately within 30 min [380]. Moreover, cucurbitacin B displayed a high tissue-to-plasma ratio, accumulating around 10-fold in several organs. The maximum accumulation of cucurbitacin was observed in the lungs, spleen, and kidneys, followed by the liver, stomach, and small intestine, and then the brain and heart [381]. The intake of 8 mg/kg cucurbitacin B by Wistar rats resulted in a peak drug concentration (Cmax) of 34.16  ±  2.91 ng/L [382].

As mentioned earlier, cucurbitacin B in its pure form had a median lethal dose of ~5 mg/kg (oral route) and 1 mg/kg (intraperitoneal) in mice, 0.5 mg/kg (intravenous) in rabbits, and 0.32 mg/kg (intravenous) in felines. Cucurbitacin B in the form of tablets has been used in China as an adjuvant for the treatment of chronic hepatitis and liver cancer since the 1980s (reviewed in [383]). A number of clinical studies revealed that cucurbitacin B -augmented the overall survival time in the hepatic cancer patients’ cohort, concomitantly decreasing the hepatitis-associated clinical symptoms [384].

As far as we are aware, no specific clinical studies on cucurbitacin safety profiling in humans have been described in the literature so far. No information on its acute and prolonged toxicity is available either. Thus, intensive preclinical and clinical studies are required to establish the safety profile and optimal doses of cucurbitacin B and other cucurbitacins in mammals, including humans.

***Betulinic acid.*** Due to its triterpene nature, betulinic acid has a poor bioavailability. Intraperitoneal injection of 500 mg/kg betulinic acid in the skin of mice resulted in Cmax 300.9 µg/mL, a half-life of 11.8 h, with significant accumulation in the ovary, spleen, mammary gland, uterus, bladder, lymph node, and liver [385].

To improve bioavailability, different BA delivery systems were created, including carbon nanotubes, magnetic and polymeric nanoparticles, conjugates, nanoemulsions, liposomes, and cyclodextrins (reviewed in [281]). Thus, Saneja and colleagues have developed BA-monomethoxy polyethylene glycol (mPEG) conjugate, which significantly improved BA solubility and antitumor efficiency. BA-mPEG internalized and induced apoptosis in hepatic cancer cells and was significantly superior in the reduction of tumors in Ehrlich ascite carcinoma mice in comparison with unconjugated BA [386]. In addition, different derivates of BA have been synthetized and evaluated [387,388]. For instance, modifications at positions C-3, C-20, and C-28 can improve water solubility without affecting its pharmacological activity [282].

According to several studies, BA has no significant adverse effects, demonstrating selective cytotoxicity against cancer cells [282]. It has been studied in several clinical trials [387]; however, its clinical studies are limited first of all due to its poor water solubility. As BA is a very promising antineoplastic compound, we are waiting for clinical trials involving nanoformulated BA-based therapeutics.

***Ginsenosides.*** Ginsenosides are characterized by the complex biotransformation during and after their absorption. Generally, they have low aqueous solubility, poor membrane permeability, and metabolic instability. Thus, their bioavailability is low and is usually less than 10% (ranging from 0.3 to 25%) (reviewed in [389]). Li and colleagues have shown that after oral administration of ginsenosides Rg1, Rb1, and Rd to rats, their highest level was detected in the liver, lung, kidney, and spleen [390].

Micronization of ginsenoside Rh2 increased its bioavailability to the level of 32% [391]. Moreover, piperine may be an efficient enhancer of Rh2 bioavailability [392]. Moreover, a plethora of nanoformulation technologies are applied to enhance ginsenosides’ bioavailability and transform them into antitumor therapy [393].

In a randomized clinical trial, the volunteers received either red ginseng extract or ginsenoside compound K (CK) with one conjugated glucose molecule (CK-30)—the fermentation product of ginsenoside CK. When compared with the red ginseng extract, the CK-30 displayed a 118.3-fold increase in Cmax [394].

Different studies have shown that ginsenosides are well tolerated and have a safety profile [395,396,397]. According to Song et al., the daily intake of 2 g of Korean red ginseng for 24 weeks is safe [398].

## 7. Conclusions and Future Perspectives

In the present review, we discussed the anticancer properties and their mechanisms of action for several natural compounds isolated from plants. Based on their wide-spread use and multitarget specificity, these compounds may have a great potential to become novel antineoplastic therapeutics. As mentioned earlier, all of them target multiple biochemical and signaling pathways and meet the criterion of multitarget therapeutics. Moreover, beyond the antitumor activity, all of these compounds display different beneficial pharmacological properties in healthy tissues, including anti-inflammatory, cardio-, neuro-, hepatoprotective, hypoglycemic, and more. These properties are of great value to cancer patients undergoing chemotherapeutic intervention because they inevitably will face the harmful off-target effects of chemotherapy, and these natural compounds may help negate the unwanted consequences.

It can be inferred from the above studies that many of these compounds can sensitize cancer cells to various therapeutics. Another possible approach is the combination of compounds described to enhance their negative impact on multiple metabolic and signaling pathways, which drive the development of malignancies.

One of the main challenges to translating natural compounds to clinics is their low bioavailability. However, there are numerous successful efforts described in the literature to increase their bioavailability by nano-delivery systems or bioenhancers. Obviously, further developments in this direction are absolutely necessary to transform natural compounds into clinically available therapeutics.

All compounds discussed demonstrate a wide range of safety profiles. Many of them have been studied in various clinical trials and/or are approved for use as dietary supplements. However, for the majority of them, there is not enough data on their safety and dozing to become full-fledged antineoplastic drugs.

By combining the development of nano-delivery systems suitable for these compounds with comprehensive pre- and clinical studies, we would be able to unleash the great potential of natural compounds to improve our efforts in fighting against cancer.

## Figures and Tables

**Table 6:** Arctigenin-mediated impact on metabolic pathways in cancer models.

Metabolic Pathway Affected	Related Targets	Type of Neoplasia	Description	Reference
OXPHOS		Lung cancer	Inhibited mitochondrial respiration and ATP production	[251]
OXPHOS	Mitochondrial chain complexes II and IV	Pancreatic cancer	Inhibited mitochondrial chain complexes II and IV and selectively killed only the OXPHOS-dependent cancer cells	[252]
FAO	CPT1	Colitis-induced colorectal cancer mouse model	Down-regulated NLRP3 inflammasome, CPT1 and FAO in macrophages; inhibited cancer development	[254]
FAO	PPARγ and C/EBPα	Non-cancer cell model (adipocytes)	Down-regulated PPARγ and C/EBPα and FAO	[255]

**Table 10:** Ginsenosides-mediated impact on metabolic pathways in cancer models.

Metabolic Pathway Affected	Related Targets	Type of Neoplasia	Description	Reference
Glycolysis	GLUT1 GLUT4	Gastric cancer	Rg3 down-regulated GLUT1/4 and VEGF both in vitro and in vivo	[311]
Glycolysis	GLUT1 HK2 PFKL PKM2 LDHA	Esophageal carcinoma	Rh4 reduced the number of glycolytic enzymes	[313]
Glycolysis	GLUT1 PKM2 LDHA	Non-small cell lung cancer	Rh2 STAT3/c-Myc axis, which reduced GLUT1, PKM2, LDHA, and suppressed EMT	[314]
Glycolysis	HK2 PKM2 c-Myc	Hepatocellular carcinoma	Compound K (CK) attenuated the AKT/mTOR/c-Myc axis, leading to HK2 and PKM2 down-regulation	[315]
Glycolysis	HIF1α GLUT1, HK2, LDHA	Hepatocellular carcinoma	Compound K (CK) down-regulated HIF1α, GLUT1 and key glycolytic enzymes under hypoxia	[316]
Glycolysis	HIF1α HK2	Ovarian cancer	Rh3 inhibited glycolysis thought DNMT3A-mediated DNA methylation in promoter region of miR-519a-5p, which targets HIF1α	[317]
Glutamine metabolism	c-Myc GLS1	Hepatocellular carcinoma	Rk1 suppressed ERK/c-Myc/GLS1 axis	[318]
Glutamine metabolism	c-Myc ASCT2 GLS1 GLUD1	Triple-negative breast cancer	Compound K (CK) inhibited glutaminolysis; decreased glutamate, proline, aspartate, asparagine, ATP, and glutathione production	[319]
